# Characterization of intestinal mononuclear phagocyte subsets in young ruminants at homeostasis and during *Cryptosporidium parvum* infection

**DOI:** 10.3389/fimmu.2024.1379798

**Published:** 2024-05-02

**Authors:** Ambre Baillou, Florian Tomal, Thierry Chaumeil, Céline Barc, Yves Levern, Alix Sausset, Tiffany Pezier, Julie Schulthess, Pauline Peltier-Pain, Fabrice Laurent, Sonia Lacroix-Lamandé

**Affiliations:** ^1^ Unité Mixte de Recherches (UMR)1282 Infectiologie et Santé Publique, INRAE Centre Val de Loire, Université François Rabelais de Tours, Nouzilly, France; ^2^ Phileo by Lesaffre, Marcq-en-Barœul, France; ^3^ Unité Expérimentale (UE)1277 Plateforme d’Infectiologie Expérimentale (PFIE), INRAE Centre Val de Loire, Nouzilly, France

**Keywords:** mononuclear phagocytes, dendritic cells, ruminants, intestine, *Cryptosporidium parvum*

## Abstract

**Introduction:**

Cryptosporidiosis is a poorly controlled zoonosis caused by an intestinal parasite, *Cryptosporidium parvum*, with a high prevalence in livestock (cattle, sheep, and goats). Young animals are particularly susceptible to this infection due to the immaturity of their intestinal immune system. In a neonatal mouse model, we previously demonstrated the importance of the innate immunity and particularly of type 1 conventional dendritic cells (cDC1) among mononuclear phagocytes (MPs) in controlling the acute phase of *C. parvum* infection. These immune populations are well described in mice and humans, but their fine characterization in the intestine of young ruminants remained to be further explored.

**Methods:**

Immune cells of the small intestinal Peyer’s patches and of the distal jejunum were isolated from naive lambs and calves at different ages. This was followed by their fine characterization by flow cytometry and transcriptomic analyses (q-RT-PCR and single cell RNAseq (lamb cells)). Newborn animals were infected with *C. parvum*, clinical signs and parasite burden were quantified, and isolated MP cells were characterized by flow cytometry in comparison with age matched control animals.

**Results:**

Here, we identified one population of macrophages and three subsets of cDC (cDC1, cDC2, and a minor cDC subset with migratory properties) in the intestine of lamb and calf by phenotypic and targeted gene expression analyses. Unsupervised single-cell transcriptomic analysis confirmed the identification of these four intestinal MP subpopulations in lamb, while highlighting a deeper diversity of cell subsets among monocytic and dendritic cells. We demonstrated a weak proportion of cDC1 in the intestine of highly susceptible newborn lambs together with an increase of these cells within the first days of life and in response to the infection.

**Discussion:**

Considering cDC1 importance for efficient parasite control in the mouse model, one may speculate that the cDC1/cDC2 ratio plays also a key role for the efficient control of *C. parvum* in young ruminants. In this study, we established the first fine characterization of intestinal MP subsets in young lambs and calves providing new insights for comparative immunology of the intestinal MP system across species and for future investigations on host–Cryptosporidium interactions in target species.

## 1 Introduction

Cryptosporidiosis is an intestinal zoonosis caused by the infection with the Apicomplexa parasite *Cryptosporidium* spp. It represents a leading cause of mild to severe diarrhea worldwide, which is self-limiting in immunocompetent individuals but one of the most lethal pathogens for malnourished children in low- and middle-income countries (1, 2). In livestock, the most sensitive hosts are young ruminants (calves, lambs, and goat kids) because of the immaturity of their intestinal immune system, with *C. parvum* as the major responsible species (3). In particular, *C. parvum* is the main cause of diarrheal enteric diseases in young calves in France and Great Britain, ahead of *Escherichia coli* and rotavirus infections (4). Responsible for aqueous diarrhea, dehydration, weight loss, growth retardation, and death of animals in the most severe cases, and in the absence of a fully effective treatment, cryptosporidiosis leads to significant human and animal health, environmental, and economic problems (4, 5).

It is now well-established that the acute phase of *C. parvum* infection is mainly controlled by the innate immune response triggered by the invasion of intestinal epithelial cells (6). Notably, we demonstrated the importance of CD11c^+^MHCII^+^ intestinal mononuclear phagocytes (MPs) in controlling the early stage of infection in the murine model, and more precisely those of type 1 conventional dendritic cells (cDC1) (7, 8). More recently, it was demonstrated that during infection, the epithelial-derived IL18 synergized with IL12 produced by activated dendritic cells (DCs) to stimulate innate lymphoid cell 1 (ILC1) production of IFNγ required for early parasite control (9). These immune populations are well-described in mice and humans, but their fine characterization in young ruminants, which are the targets of the parasite in livestock, remains to be further explored together with the evaluation of their relative importance in the control of cryptosporidiosis. Comparative studies have allowed the establishment of a common inter-species classification of the MP system based on phenotypic and functional equivalencies of monocytic and DC subsets from various species (mouse, human, pig, chicken, cow, and sheep, at various stages of progress depending on the species) and tissues (10–12). In addition, single-cell RNA sequencing analyses can now provide a step further in the definition of their lineage and fine characterization of subsets and diversity of cell states (13–17).

*C. parvum* multiplies in the distal part of the small intestine of its hosts. Young ruminants harbor a peculiar continuous large ileal Peyer’s patch (IPP) at birth that extends throughout the ileum, together with more classical isolated multiple jejunal Peyer’s patches (JPPs) (18). Peyer’s patches (PPs) are intestinal lymphoid tissues representing major inducing sites of immune responses, developed but still immature at birth in ruminant species (19). The IPP acts as a primary lymphoid organ for the generation of B-cell repertoire of the intestine and peripheral organs (lymph nodes and spleen), while JPPs are recognized as secondary lymphoid organs for the activation of lymphocytes and induction of mucosal immune responses (18, 20, 21). Regarding their functions, the IPP undergoes involution with age, from 3 to 4 months old for lambs, until it almost completely regressed, whereas JPPs are functionally maintained in adulthood (19, 22).

In this study, in order to better define intestinal MPs of young ruminants, we performed phenotypic and transcriptomic analyses of macrophages (MAC) and DC subsets from lymphoid and non-lymphoid intestinal tissues of lambs and calves at homeostasis and during lifetime progression for sheep. These cells were also studied in the intestine of experimentally *C. parvum*-infected lambs and calves.

## 2 Materials and methods

### 2.1 Ethics statement

Animal needs were met in accordance with the European Community Council Directive 2010/63/EU (Decree: 2013-118 01/02/2013). The experimental facilities had received authorization to house experimental animals from the local bureau of veterinary services (Indre-et-Loire, France, authorization no. D 37-175-3), and all the experimental procedures were approved by the Val de Loire Ethics Committee (CEEA19) (authorization no. APAFlS#21604-201907250902391 v2 and #21515-2019071714558143 v2). All animal experimentations have been performed in the Infectiology of Farm, Model and Wildlife Animals Facility (PFIE, Centre INRAE Val de Loire: doi.org/10.15454/1.5572352821559333E12; a member of the National Infrastructure EMERG’IN: doi.org/10.15454/90CK-Y371). All the personnel involved had dedicated training in animal care, handling, and experimentation, as required by the French Ministry of Agriculture.

### 2.2 Lambs and calves

Pré-Alpes (*C. parvum* infection experiments) and Ile de France lambs (cell phenotyping) were respectively provided by the sheep farming of the PFIE and of the Animal Physiology Experimental Unit (UE PAO, Centre INRAE Val de Loire). Prim’Holstein calves with excellent health status were from approved local cattle breeders. For the intestinal MP cell subset characterization, 8-day-old, 10-day-old, and 4-month-old lambs and 3-year-old sheep were used. For the *C. parvum* lamb infection experiments, six or seven lambs per group (a non-infected group and an infected group) were enrolled at 1 day of age, after colostrum intake from their dams. For the calf study, we benefited from concomitant experiments enrolling young calves to collect intestinal samples from four 10-day-old non-infected animals and from three 12- to 13-day-old infected animals, inoculated at day 1 of age by oral route with 8 × 10^6^ oocysts of *C. parvum* INRAE strain resuspended in water. The number of animals used in each condition is summarized in **Table 1**.

**Table 1:** Number of animals used in each experimentation.

Experimentation with ovines					
*At homeostasis (with Ile de France sheep breed)* At different ages		1 day (*n* = 8)	10 days (*n* = 6)	4 months (*n* = 5)	3 years (*n* = 3)
*Infection experimentations (with Pré-Alpes lambs)* Experimentation no. 1 Experimentation no. 2		*n* = 6–7 non-infected; *n* = 6–7 *C. parvum* infected (6 dpi) *n* = 6–7 non-infected; *n* = 6–7 *C. parvum* infected (12 dpi)			
Experimentation with bovines (Prim’Holstein)					
Non-infected *C. parvum* infected (12–13 dpi)	*n* = 10 *n* = 3				

### 2.3 *C. parvum* parasite and infection of lambs

#### 2.3.1 Experimental design

Studies were composed of two groups of six lambs each: one control group with non-infected lambs and one *C. parvum*-infected group. Lambs from the infected group were inoculated by oral route with 2 × 10^6^ oocysts of *C. parvum* in water at 3 or 4 days of age. The *nluc*-INRAE transgenic strain of *C. parvum* (23), which expresses the nanoluciferase enzyme, was used in the study to quantify parasite load in the intestine. Two independent experimentations were realized according to the timing of interest, i.e., at the peak or during the resolution of infection, corresponding respectively to 6 and 12 days post-infection (dpi). Lambs were fed *ad libitum* with unlimited access to milk (Colostromix^®^, Technovet Eurotonic) with milk feeders disposed in each pen. After euthanasia of the animals at 6 or 12 dpi, intestinal tissue samples were collected for analysis of mRNA expression by real-time RT-PCR or flow cytometry analysis.

#### 2.3.2 Parasite burden monitoring

Feces were collected daily from 3 to 6 or 12 dpi in order to follow the lamb parasite excretion. Parasite loads in feces were determined by counting *C. parvum* oocysts in Sheather’s solution using a Thoma cell counting chamber with an optical microscope. Fecal samples differing on their diarrheal consistency, we normalized parasite loads with the moisture rate of feces, calculated by comparing the weight of each fecal sample before and after 48 h of drying at +60°C. Therefore, results were expressed as oocysts per gram of dry feces. In addition, the level of infection in the lamb intestine according to the intestinal segment was evaluated by measuring luciferase activities of the *C. parvum* transgenic strain in duodenum, JPP, distal jejunum, proximal and distal IPP, cecum, and colon tissues, at 6 dpi, as previously described (24).

#### 2.3.3 Clinical sign assessment

The health status of animals was evaluated by assessing their clinical signs all along the experimentations. Each lamb was weighed each day in order to follow its daily weight gain (DWG). The general status (depression signs), dehydration status (based on skin fold persistence and eyes appearance), temperature score (based on variation from physiological temperature), and fecal index (consistency of feces related to diarrhea, ranging from 0 to 2, with 0 corresponding to normal feces, 1 to semi-liquid feces, and 2 to liquid feces) were evaluated daily by skilled animal technicians. The combination of these four parameters established a global score, representative of the health status of animals. The limit point, according to ethics and animal welfare statements, was considered to be reached if their global score was superior to 7 during two consecutive days. Of note, the limit point was never reached by any of the animals during the experiments.

### 2.4 Isolation of intestinal cells from tissues

Tissues from IPP, JPP, and distal jejunum of lambs and calves were sampled and washed with PBS. Intestinal mucosa was isolated from the muscularis by scraping with a scalpel and enzymatically dissociated in culture medium (RPMI; 10% FBS; 100 U/mL penicillin; and 100 µg/mL streptomycin) with 0.02 MU/mL DNase I, 0.9 U/mL dispase II, and 0.8 mg/mL collagenase I (Sigma-Aldrich) for 30 min at +37°C under agitation at 700 rpm. Resulting intestinal cellular suspensions were filtered on 100-µm cell strainers and washed with culture medium. Red blood cells were removed by a 5-min incubation at room temperature (RT) with a lysis buffer (Sigma-Aldrich). A Ficoll gradient (Sigma-Aldrich) was performed and centrifuged at 2,000×*g* for 30 min at RT. Cells from the interphase were washed with culture medium to obtain the total isolated intestinal cells.

### 2.5 Flow cytometry and cell sorting

Isolated cells from IPP, JPP, and distal jejunum were resuspended as 2 × 10^6^ cells in FACS buffer (PBS; 2% FBS; 2 mM EDTA). Cells were next incubated for 15 min in Fc block (FACS buffer; 2% ovine or bovine serum according to the experiment). Staining was then performed by incubation with a viability marker (Zombie Aqua™ Fixable Viability Kit, BioLegend) and species cross-reacting antibodies are listed in **Table 2**. The immunostaining of the cells with the antibodies was carried out in three steps of 30 min in the dark at 4°C+, each of them being separated with a series of three washes with FACS buffer. During the first step, the cells were stained with the mouse IgG1 anti-bovine CD14. In the second step, cells were incubated with the secondary antibody anti-mouse IgG1 (rat IgG1, BV711, clone A85-1, BD Biosciences) and a mix of primary antibodies composed of the mouse IgM anti-CD11c and the chicken anti-human Cadm1. In the last step, the cells were incubated with the secondary antibodies [anti-mouse IgM (rat IgG2a, APC-Cy7, clone RMM-1, BioLegend) and anti-chicken IgY (goat IgG, Alexa Fluor 647, polyclonal, Abcam)], the directly conjugated primary antibodies (anti-ovine MHCII-RPE, anti-bovine CD11b-FITC, and the anti-bovine CD172α^−^ RPE-Cy5), and the Aqua Zombie viability probe. The anti-bovine CD172α was stained with the LYNX Rapid RPE-Cy5 Antibody Conjugation Kit, according to the manufacturer’s instructions (Bio-Rad). After staining, cells were either fixed with Stabilizing Fixative buffer (BD Biosciences) or conserved in PBS respectively for flow cytometry analyses with the LSRFortessa X-20 flow cytometer (Becton Dickinson) or for cell sorting with the MoFlo Astrios EQ cell sorter (Beckman Coulter). Unstained cells and matched isotype controls were used to define gating thresholds. Gating on forward scatter (FSC) and side scatter (SSC) parameters was first used to exclude cell debris (i.e., FSC^low^ and SSC^low^ events), and viable cells were next selected by exclusion of dead cells (i.e., viability marker-positive cells). Finally, among CD11c^+^MHCII^+^ cells gated as MP cells, the following four subsets were discriminated and sorted: CD14^+^CD172α^+^Cadm1^int^CD11b^+^ cells (subset 1), CD14^−^CD172α^−^Cadm1^+^CD11b^−^ cells (subset 2), CD14^−^CD172α^+^ Cadm1^int^CD11b^+^ cells (subset 3), and CD14^−^CD172α^+/−^Cadm1^−^CD11b^−^ cells (subset 4). The complete gating strategy is available in **Supplementary File 1**.

**Table 2:** List of antibodies.

Antibody	Specificity	Isotype	Clone	Source	Cross-reactivity	Reference
Anti-ovine MHC II-RPE	MHCII	Mouse IgG1	28.1	Bio-Rad	Cattle	Datasheet MCA2225PE
Anti-bovine CD172α	CD172α	Mouse IgG1	DH59B	Bio-Rad	Sheep	Datasheet MCA6079
Anti-bovine CD11b-FITC	CD11b	Mouse IgG2b	CC126	Bio-Rad	Sheep	Datasheet MCA1425F
Anti-bovine CD11c	CD11c	Mouse IgM	BAQ153A	Bio-Rad	Sheep	Datasheet MCA6084
Anti-bovine CD14	CD14	Mouse IgG1	CAM36A	Bio-Rad	Sheep	Datasheet MCA6085
Anti-human Cadm1	Cadm1	Chicken IgY	9D2	MBL	Sheep	10.4049/jimmunol.1000824.

### 2.6 Analysis of mRNA expression by real-time RT-PCR

For mRNA expression analyses in intestinal MP subsets, RNA were recovered from sorted cells with a lysis buffer [RNase/DNase-free water; 0.01 M DTT; 1% Tween 20; 0.83 U/µL RNAseOUT™ recombinant ribonuclease inhibitor (Invitrogen)] and immediately reverse-transcribed as previously described (25). For total intestinal tissue samples, RNA extractions were performed after homogenization in TRIzol (Invitrogen) with an Ultra-turrax and processed according to the manufacturer’s recommendations. Total RNA was reverse-transcribed with the iScript RT SuperMix kit (Bio-Rad). cDNA from sorted MP cell subsets and intestinal tissues were then amplified by quantitative real-time PCR (qPCR) with the FLUIDIGM^®^ method using an IFC Controller HX and BioMark™ HD thermal cycler (Fluidigm), according to the manufacturer’s instructions. We used a dynamic array IFC 96 × 96 (Fluidigm) to analyze the expression of a large panel of genes, whose primer sequences are listed in **Supplementary Table 1** and previously published (26, 27). Data of mRNA gene expression were analyzed with the Real-Time PCR Analysis software (Fluidigm). Following normalization with three reference genes (*HPRT*, *GAPDH*, and *ACTB*), results were expressed as either 2^-ΔCt^ ratio (comparison of intestinal immune responses between non-infected and *C. parvum*-infected lambs) or relative gene expression (RT-qPCR analysis of sorted MP cells).

### 2.7 Single-cell RNA sequencing with the 10X Genomics Chromium protocol

Mononuclear phagocytes were isolated as previously described from the IPP of a 10-day-old lamb and were analyzed with the 10X Genomics Chromium single-cell technology. Library was prepared according to the instructions of the 10X Genomics Single-Cell 3′ Reagent Kit v3.1 User Guide. Briefly, in order to sort viable CD11c^+^MHCII^+^ cells, total isolated intestinal cells were stained with a viability marker (Zombie Aqua™ Fixable Viability Kit, BioLegend) and with anti-bovine CD11c anti-bovine MHCII antibodies. Sorted MP cells were collected in RPMI containing 10% FBS, and 10,000 cells were used for library construction. First, MP suspension was mixed with a Master Mix containing reverse transcription (RT) reagents and loaded onto Chromium Next GEM Chip G, together with gel beads [containing an Illumina sequencing primer site, a 10X Genomics barcode, randomers called unique molecular identifiers (UMIs), and a poly-dT primer] and partitioning oil for the emulsion reaction. Nanoliter-scale Gel Beads-in-Emulsion (GEMs) were next generated with Chromium, such that each single cell was associated with a specific barcode and each of its transcripts was labeled with a UMI. After RT of mRNAs, the whole generated cDNAs were pooled following GEMs disruption/breakage, purified with Dynabeads^®^ MyOne™ SILANE (Thermo Fisher Scientific), amplified by PCR, and size-selected with SPRIselect (Beckman Coulter). The quality and quantity of cDNA were then assessed with an Agilent 2100 Bioanalyzer System with a High-Sensitivity DNA kit, according to the manufacturer’s instructions. Finally, the sequencing library construction was performed from cDNA and involved steps of enzymatic fragmentation, end repair, A-tailing, adaptor ligation, double-sided size selections with SPRIselect (Beckman Coulter), and sample index PCR, notably to add P5 and P7 primers used in Illumina amplification. Illumina paired-end sequencing of the library was realized at Integragen, using NovaSeqTM 6000 S2 systems. The read setup was as follows: read 1: 28 cycles, i7 index: 10 cycles, i5: 10 cycles, and read 2: 91 cycles.

### 2.8 Single-cell RNA-seq data analysis and visualization

The raw scRNA-seq FASTQ files were processed using Cell Ranger software (version 6.0.2, 10X Genomics) and aligned to the ARS-UI_Ramb_v2.0 reference genomes. Bam files and filtered expression matrices were generated using the “cellranger_count” pipeline. Matrices from Cellranger were further analyzed into R (version 4.10) using the Seurat R package (version 4.21) (28, 29). To exclude low-quality doublet and dead cells, cells that expressed less than 600 and more than 6,000 genes and had greater than 5% of mitochondrial genes were filtered out the analysis. Data were normalized using the default method (LogNormalize) in the Seurat package and highly variable genes were identified. Next, a linear dimensional reduction was performed on scaled data [principal component analysis (PCA)]. For further downstream analysis, the optimal number of principal components was identified by the elbowplot method. The first 20 principal components were selected for clustering the cells by the Louvain algorithm with 0.5 resolution. UMAP was employed for non-linear dimensional reduction and cluster visualization. To annotate clusters, the top 10 differentially expressed genes (DEGs) for each cluster (based on log2 fold change) were evaluated with the “FindAllMarkers” function and displayed on heatmaps or dot plots. Differential expression was also performed between two individual clusters to determine cell populations more accurately. Single-cell RNA-seq data were visualized in feature plots, violin plots, dot plots, and heatmaps using Seurat or ScCustomize R packages (version 0.7.0) (28, 30).

### 2.9 Statistical analyses

Graphical representations were created using the GraphPad Prism software (version 6.0) for histograms, the Kaluza Analysis software (version 2.1) for flow cytometry gating panels, the RStudio software (version 2023.03.0) for PCA, and heatmap representations and the Microsoft Excel program for pie charts. All statistical analyses were performed with the GraphPad Prism software (version 6.0). The appropriate statistical tests and post-hoc testing selected for each experiment are specified in figure legends.

## 3 Results

### 3.1 Identification of intestinal mononuclear phagocyte subsets in neonatal lambs and calves

The phenotypic characterization of intestinal MPs in 10-day-old neonatal ruminants was performed with flow cytometry analyses. Cells isolated from the IPP of young lambs and calves were analyzed for their cell surface expression of several markers used to distinguish MAC and DC subsets in various species (CD11c, MHCII, CD14, CD172α, CD11b, and Cadm1).

**Figure 1:**
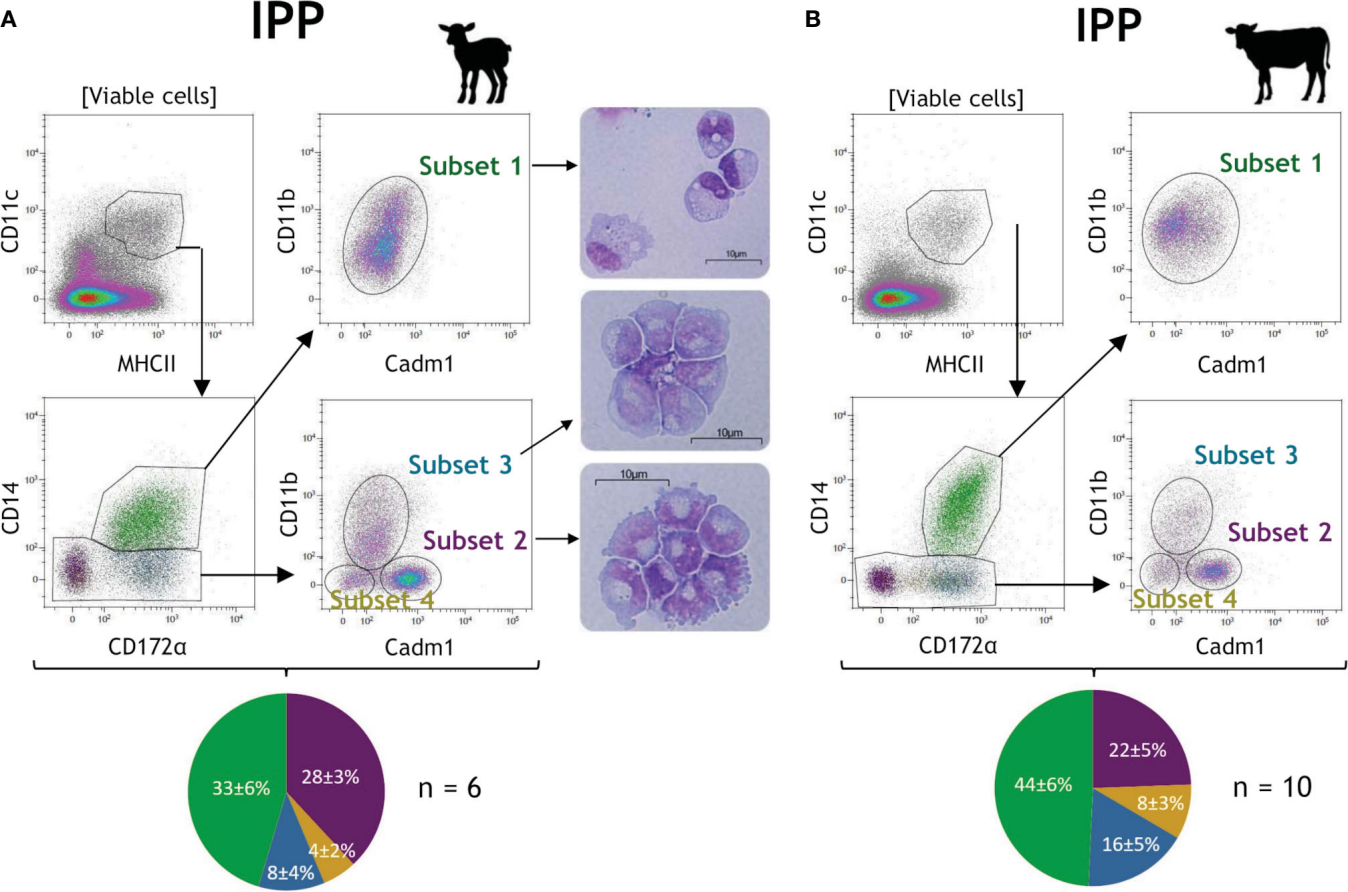
Identification of neonatal intestinal mononuclear phagocytes in the ileal Peyer’s patch of lambs and calves. Intestinal tissues from the ileal Peyer’s patch (IPP) were recovered from 10-day-old lambs (*n* = 6) and calves (*n* = 10). Mechanical and enzymatic dissociations were performed to obtain total isolated intestinal cells, then stained for flow cytometry. Representative gating for mononuclear phagocytes (MP) subsets are shown for the IPP of lamb **(A)** and calf **(B)**. Following exclusion of dead cells, CD11c^+^MHCII^+^ cells were gated to select total MP and then four subsets were distinguished based on surface expression of CD14, CD172α, CD11b, and Cadm1: CD14^+^CD172α^+^Cadm1^int^CD11b^+^ (subset 1, green), CD14^−^CD172α^−^Cadm1^+^CD11b^−^ (subset 2, purple), CD14^−^CD172α^+^Cadm1^int^CD11b^+^ (subset 3, blue), and CD14^−^CD172α^+/-^Cadm1^−^CD11b^−^ (subset 4, yellow). Subsets 1, 2, and 3 of MP from the IPP of a 10-day-old lamb were cell sorted by flow cytometry, cytospined, and stained with hematoxylin and eosin. Pictures of the three subsets of MP were obtained by optical microscopy (**A**, right panel). Pie charts represent the cell proportions in percentage of each intestinal MP subset among viable CD11c^+^MHCII^+^ cells (mean ± SD) for lambs (*n* = 6; **A**, bottom panel) and calves (*n* = 10; **B**, bottom panel).

After the exclusion of dead cells, CD11c^+^MHCII^+^ cells were initially gated to select conventional DC (cDC), MAC, and newly recruited monocytes in the intestinal tissue, while excluding pDC with little or no expression of CD11c (12) (**Figure 1**). Then, with expression of CD14 being widely used to differentiate MAC/monocytes from DCs, CD14^+^ cells were associated with putative MAC/newly recruited monocytes, and CD14^−^ cells were associated with putative cDCs. Finally, based on surface expression of CD14, CD172α, CD11b, and Cadm1, we distinguished one subset of putative MAC with a CD14^+^CD172α^+^Cadm1^int^CD11b^+^ phenotype (subset 1, green) and three subsets of putative cDC with CD14^−^CD172α^−^Cadm1^+^CD11b^−^ (subset 2, purple), CD14^−^CD172α^+^Cadm1^int^CD11b^+^ (subset 3, blue), and CD14^−^CD172α^+/−^Cadm1^−^CD11b^−^ phenotypes (subset 4, yellow), in both species (**Figure 1**). Furthermore, the combined expression of CD14, CD172α, and CD11b observed for subset 1 was described as specific for MAC/monocyte populations (31). Microscopic observation of the lamb cell morphology of subsets 1, 2, and 3 supports these assumptions, with subset 1 showing MAC-specific granules and intracellular vacuoles, while subsets 2 and 3 are somewhat smaller, with a larger nucleus and displaying DC-specific dendrite/pseudopod-like structures, as previously described (32–34) (**Figure 1A**, pictures). Among DCs, high expression of Cadm1 without CD172α and CD11b is described as cDC1-specific, while the opposite is cDC2-specific (10–12), leading us to designate subsets 2 and 3 as putative cDC1 and cDC2, respectively. Subset 4 was present at a lower frequency and might represent another cDC subset.

Moreover, additional intestinal segments were investigated for MP populations in the same animals. Similar results were obtained for the distal jejunum (non-lymphoid tissue) and the JPP in both lamb and calf (**Supplementary Files 2, 3**), indicating that the four MP subsets are present in the small intestine of young ruminants, regardless of the location (ileum or jejunum) and the presence of lymphoid structure or not.

In lamb and in the distal jejunum of calf, we also observed the distinction of two subpopulations among putative MAC (subset 1), according to a high or intermediate expression of CD11b, which was not explored further in this study (**Figure 1**; **Supplementary Files 2, 3**).

Overall, subsets 1 and 2 are the most abundant cells within MPs in the intestine of lamb (according to the tissue, 24%–33% and 28%–34%, respectively) and calf (30%–44% and 22%–34%, respectively), followed by subset 3 (8%–12% for lamb and 14%–16% for calf) and the minor subset 4 (<6% for lamb and <10% for calf) (**Figure 1**; **Supplementary Files 2, 3**, pie charts). The main difference between lymphoid and non-lymphoid tissues is the lower proportion of CD11c^+^MHCII^+^ MPs in the IPP (3.4% ± 1.1% for lamb and 2.8% ± 0.8% for calf) and JPP (5.3% ± 1.0% for lamb and 5.8% ± 1.2% for calf) in comparison to the distal jejunum (12.3% ± 2.6% for lamb and 7.7% ± 1.6% for calf), which probably results from the large representation of lymphocytes in lymphoid tissues.

### 3.2 Progression of intestinal mononuclear phagocyte subsets from birth to adult age in ovines

**Figure 2:**
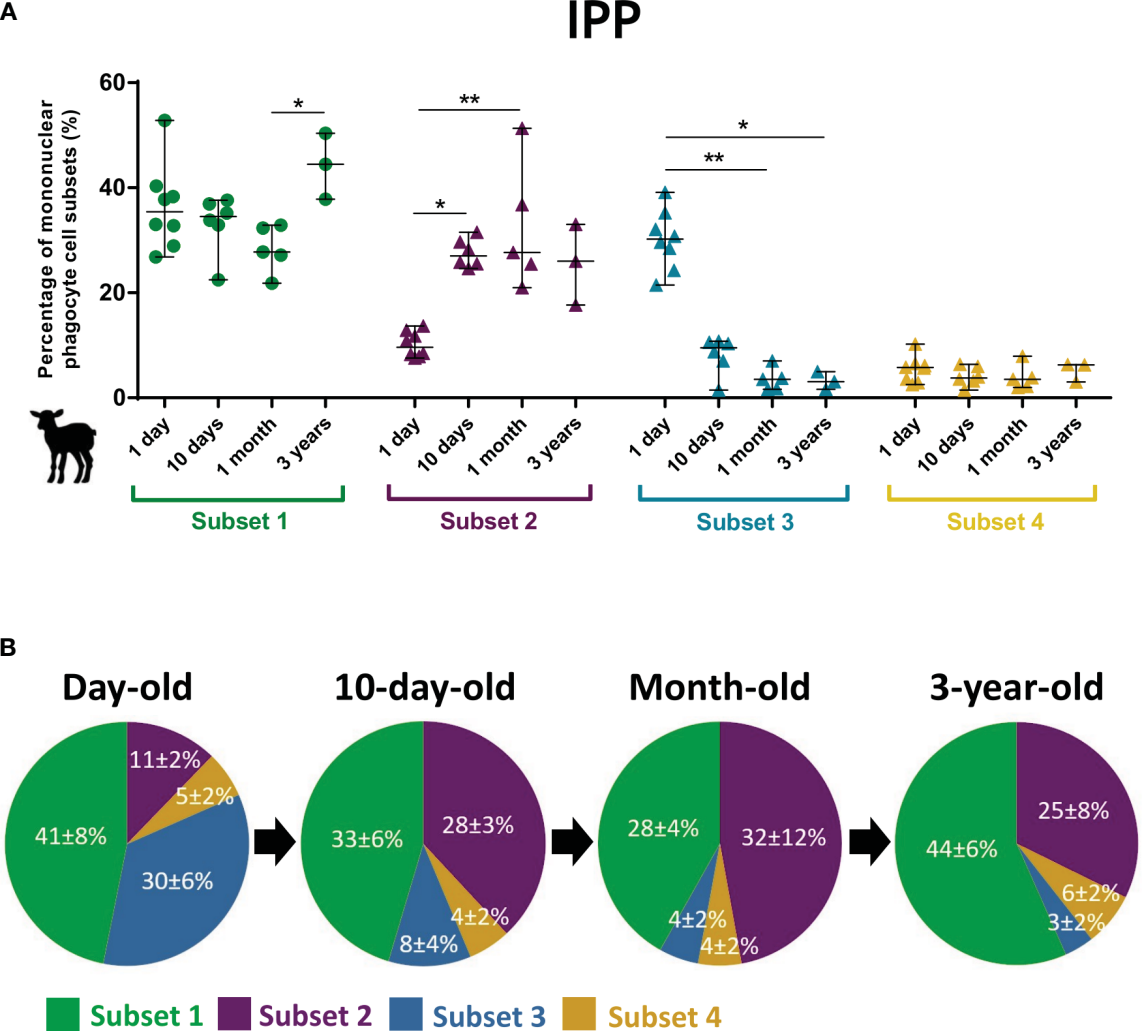
Subset proportions of intestinal mononuclear phagocytes in the lambs at different ages. Intestinal cells from the ileal Peyer’s patch were recovered from day-old (*n* = 8), 10-day-old (*n* = 6), and month-old lambs (*n* = 5) and from ileum of 3-year-old ewes (*n* = 3). Mechanical and enzymatic dissociations were performed to obtain total isolated intestinal cells, then stained for flow cytometry. Following exclusion of dead cells, CD11c^+^MHCII^+^ cells were gated to select total mononuclear phagocytes (MP), and then four subsets were distinguished based on surface expression of CD14, CD172α, CD11b, and Cadm1: CD14^+^CD172α^+^Cadm1^int^CD11b^+^ (subset 1, green), CD14^−^CD172α^−^Cadm1^+^CD11b^−^ (subset 2, purple), CD14^−^CD172α^+^Cadm1^int^CD11b^+^ (subset 3, blue), and CD14^−^CD172α^+/-^Cadm1^−^CD11b^−^ (subset 4, yellow). **(A)** Cell proportions of ileal MP subsets according to the age of animals, expressed in percentage of MP (median ± range) with each point corresponding to one animal. **(B)** Pie charts of cell proportions of ileal MP subsets by age group, expressed in percentage of MP (mean ± SD). Statistical analyses were performed using Kruskal–Wallis non-parametric test followed by Dunn’s multiple comparison test to compare the medians of MP subsets between age groups; statistical significance was determined by a *p*-value < 0.05 (*p < 0.05, **p < 0.01).

The intestinal immune system develops and matures with age with a rapid acceleration after birth upon contact with microbiota and alimentary antigens. A peculiarity of young ruminants is their large IPP, which starts to regress from approximately 3 months old until it disappears as an adult (19). *C. parvum* infection affects particularly the ileum of neonatal lambs while adults are strongly resistant to the infection (35, 36). Thus, the ovine intestinal MP subsets from birth to adult age were analyzed by flow cytometry. In IPP, a striking feature is the tripling of the proportion of putative cDC1 subset 2 within the 10 days after birth while those of putative cDC2 subset 3 decreases by almost 10-fold in the first month (**Figure 2**). Similar observations, albeit at a lower magnitude, were found in the other intestinal segments analyzed, i.e., the distal jejunum (twofold increase and decrease for putative cDC1 subset 2 and putative cDC2 subset 3, respectively) and JPP (almost threefold increase and fourfold decrease for subsets 2 and 3, respectively) (**Supplementary File 4**). This suggests that these changes in the proportion of putative cDC1 and cDC2 occur in both lymphoid and non-lymphoid structures in the small intestine of young lambs. At 3 years of age, the proportion of CD11c^+^MHCII^+^ was higher than in young animals due to the decrease in the presence of lymphocytes related to the involution of the IPP (data not shown).

### 3.3 Characterization of intestinal mononuclear phagocyte subsets in lambs and calves

Intestinal MP subsets primarily identified based on cell surface markers were further characterized by transcriptomic analyses following cell sorting. We studied the expression of hallmark genes belonging to the conserved transcriptomic signatures of mammalian MAC and cDC subsets across species (human, mouse, pig, chicken, cattle, and sheep) (10–12) and of genes involved in immune responses (69 genes in total listed in **Supplementary Table 1**).

**Figure 3:**
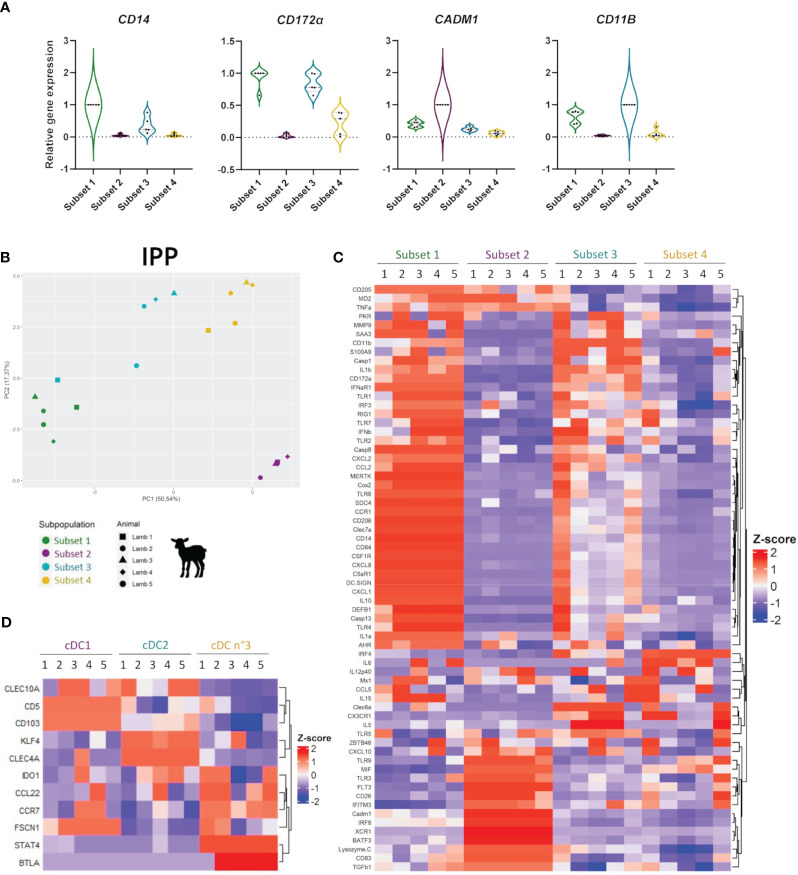
Transcriptomic analyses of mononuclear phagocyte subsets in the ileal Peyer’s patch of lamb. Gene expression in the four sorted ileal mononuclear phagocyte (MP) subsets of 10-day-old lambs (*n* = 5) was assessed by classical quantitative RT-PCR **(D)** or with the FLUIDIGM^®^ method **(A–C)**. Gene expression was defined by relative gene expression levels normalized to maximal expression across cell subsets, following normalization with three housekeeping genes (*HPRT*, *GAPDH*, and *ACTB*) and 2e^-ΔCt^ value calculation. **(A)** Expression of the genes coding for the proteins used for cell sorting (*CD14*, *CD172α*, *CADM1*, and *CD11B*) in each cell subset, represented by violin plots (median and quartiles). Each point corresponds to one animal. **(B)** Principal component analysis (PCA) represented by its first two dimensions with corresponding variances in percentage and performed on relative gene expression values. The 66 genes displayed in **(C)** were included in the PCA. Each point corresponds to the data of one animal, with one point shape per individual animal. **(C)** MP subset-specific gene transcription represented by heatmaps of the *Z*-score normalized relative gene expression values for each gene analyzed in the four MP subsets, with hierarchical clustering of genes. According to their gene expression profile, subset 1 was identified as macrophages (MAC), subset 2 as type 1 conventional dendritic cells (cDC1), subset 3 as type 2 cDC (cDC2), and subset 4 as cDC no. 3. **(D)** Transcription of a selected set of genes characterizing DC subsets represented by the heatmap of the *Z*-score normalized relative gene expression values for each gene analyzed in the three DC subsets, with hierarchical clustering of genes.

First, we observed that *CD14*, *CD172α*, *CADM1*, and *CD11B*, the four genes coding for the corresponding surface proteins used for cell sorting, showed a specific pattern of expression for each cell subset from the intestine of lambs and calves (**Figure 3A**; **Supplementary Files 5, 6**) corresponding to those at the protein level (**Figure 1**; **Supplementary Files 2, 3**).

In the IPP of lambs, the four MP subsets previously identified by flow cytometry are clearly distinguished from each other according to their gene expression profiles following PCA (PC1 = 50.54%, PC2 = 17.37%) (**Figure 3B**). The major gene expression of *CD14*, *CD64*, *C5αR1*, and *MERTK*, together with the absence of that of *FLT3* and *ZBTB46* in subset 1, confirmed their identification as MAC (**Figure 3C**). In parallel, subsets 2, 3, and 4 display co-expression of *FLT3* and *ZBTB46*, two cDC hallmark genes, with subset 2 expressing the highest level of *FLT3* (**Figure 3C**). The mutually exclusive gene expressions of *BATF3*, *XCR1*, *IRF8*, *CADM1*, *CD26*, and *TLR3* in subset 2, and of *IRF4*, *FCεR1α*, *CLEC6A*, *CLEC7A*, *CX3CR1*, *CD172α*, *CD11B*, *IL1β*, *TLR7*, and *TLR8* in subset 3 indicate that they correspond to cDC1 and cDC2, respectively (**Figure 3C**). In addition, the gene expression profiles of subsets 1 (MAC) and 3 (cDC2) are relatively close, showing the common expression of the majority of the genes studied but with different expression levels. cDC subset 4 has a more heterogeneous gene expression profile, sharing both feature gene expression of subset 2 (cDC1) (*TLR9*, *CD26*, *IL12P40*, and *MIF*) and subset 3 (cDC2) (*TLR7*, *IRF4*, *CX3CR1*, *CLEC6A*, and *CCL5*) (**Figure 3C**). Nevertheless, its high expression of *IRF4* and closer proximity to cDC2 in PCA suggests that it might be a cDC2 subpopulation or a cDC2 subset in a different state of maturation (subset 4 will now be called “cDC no. 3“). Additional key genes known to describe the heterogeneity within cDC subpopulations were therefore further analyzed. As shown in **Figure 3D**, cDC no. 3 stands out by high expression of *CCR7*, *CCL22*, *FSCN1*, and *STAT4*, which are involved in the processes of activation, maturation, and migration of DC to mesenteric lymph nodes (37–39), indicating that they may correspond to migratory DCs. This subset also highly expresses *BTLA* and *IDO1*, two genes associated with immune regulatory activities (39–41) (**Figure 3D**). These results were also obtained in the distal jejunum and JPP of lambs, confirming that this subset characterization is present in the different parts of the small intestine (**Supplementary File 5**).

In the IPP of calves, similar analyses confirm the characterization of MAC, cDC1, and cDC2 and therefore indicate that these populations might be present across ruminant species (**Supplementary File 6**). However, in calves, PCA cannot really distinguish cDC2 from cDC no. 3 (**Supplementary File 6B**).

### 3.4 Fine clustering of intestinal dendritic and monocytic cells in lamb by scRNA-seq

In order to better define DC and MAC subsets, a gene clustering was performed at the single-cell level. Alive CD11c^+^MHCII^+^ cells from the IPP of a 10-day-old lamb were sorted, and single-cell transcriptomes were obtained and processed as described in Materials and Methods. A resolution of 0.5 was chosen, resulting in 15 different clusters (**Supplementary File 7A**). We first analyzed *FLT3* and *CSF1R* gene expression in UMAP plots to identify DC and monocytic cell clusters, respectively (**Supplementary File 7B**). Few cells (no. 13), lacking both *FLT3* and *CSF1R* expression, were identified as B lymphocytes as indicated by their specific expression of *LTB, JCHAIN*, and *MS4A1* genes and were excluded from further cluster analyses (**Supplementary File 7C**; **Supplementary Table 2**).

**Figure 4:**
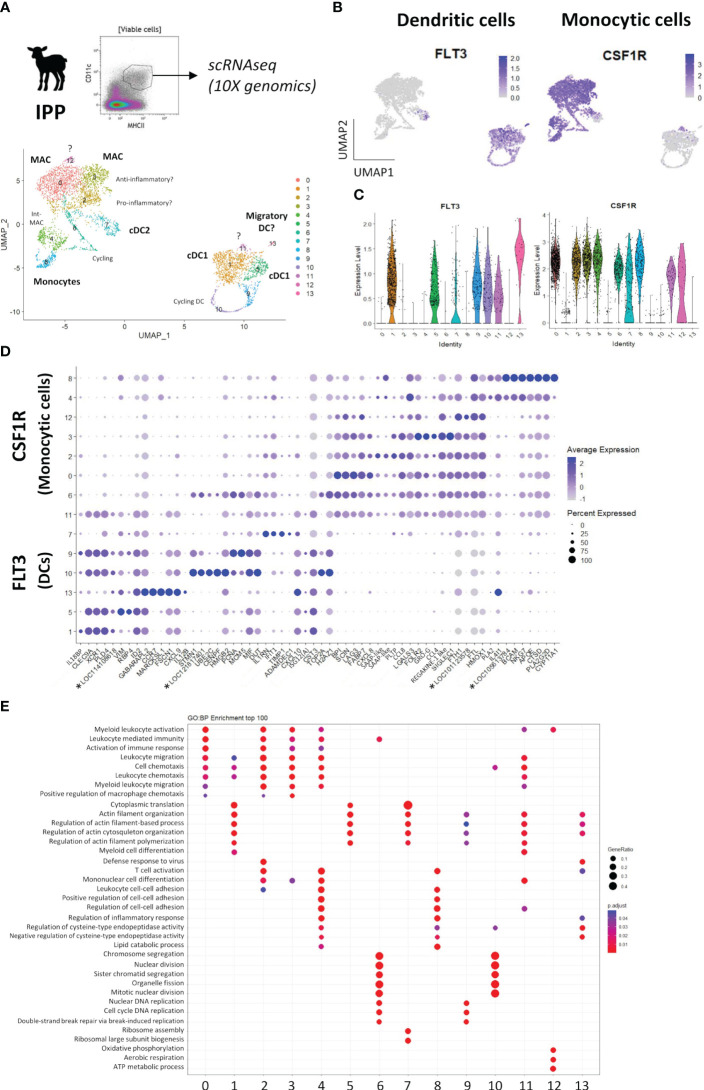
Single-cell RNA-sequencing of mononuclear phagocytes from the ileal Peyer’s patch of lamb. Mononuclear phagocytes (MP) from the ileal Peyer’s patch of a 10-day-old lamb, identified as CD11c^+^MHCII^+^ cells, were sorted by flow cytometry and subjected to 10X Genomics scRNA-seq. **(A)** Data from 10,000 MP cells were analyzed and clustering was performed with a resolution of 0.5, resulting in 14 distinct clusters visualized by a UMAP plot. DC, dendritic cells; cDC1, type 1 conventional DC; MAC, macrophages; int-MAC, intermediary differentiation stage of MAC. **(B)** UMAP plots showing the expression of *FLT3* and *CSF1R*, defining dendritic and monocytic cell clusters, respectively. Expression levels are visualized from low expression (gray) to high expression (blue). **(C)** Violin plots showing the level of *FLT3* and *CSF1R* expression across all clusters. **(D)** Dot plot showing the expression of the top 10 differentially expressed genes (lowest p_val_adj, highest avg_log2FC) for each cluster, as determined by Seurat’s FindAllMarkers function. Complete gene lists are given in **Supplementary Table 3**. **(E)** Dot plot resulting from the gene ontology (GO) enrichment analysis of the top 100 differentially expressed genes for each cluster of DC and monocytic cells (biological process), and showing the top three GO terms significantly (*p* < 0.05) enriched in c0 to c13. *Genes of unknown function.

After reclustering (**Figures 4A-C**), *FLT3*-expressing DC cells could be associated with five clusters (clusters 1, 5, 9, 10, and 13) and *CSF1R*-expressing monocytic cells could be associated with seven clusters (clusters 0, 2, 3, 4, 6, 8, and 12), with two other clusters (clusters 7 and 11) expressing both *FLT3* and *CSF1R*. In particular, c7 contained cells expressing either *FLT3* or *CSF1R* (**Figure 4B**). CSF1R-expressing clusters were also characterized by the expression of additional signature genes for monocytic cells (*CD68*, *MERTK*, *C1QA*, *C1QB*, and *C5AR1*), absent from FLT3-expressing clusters with the exception of a part of cells in c7 (**Supplementary File 7D**). Moreover, the expression of the top 10 DEGs by each cluster (lowest p_val_adj, followed by highest avg_log2FC) visualized in a dot plot for all clusters highlighted the close proximity of DC cluster 7 and cluster 11 to monocytic cells (**Figure 4D**; **Supplementary Table 3**). In addition, DC clusters displayed common enriched gene ontology (GO) terms related to cytoskeleton organization while those for monocytic cell clusters were related to leukocyte activation and chemotaxis (**Figure 4E**).

**Figure 5:**
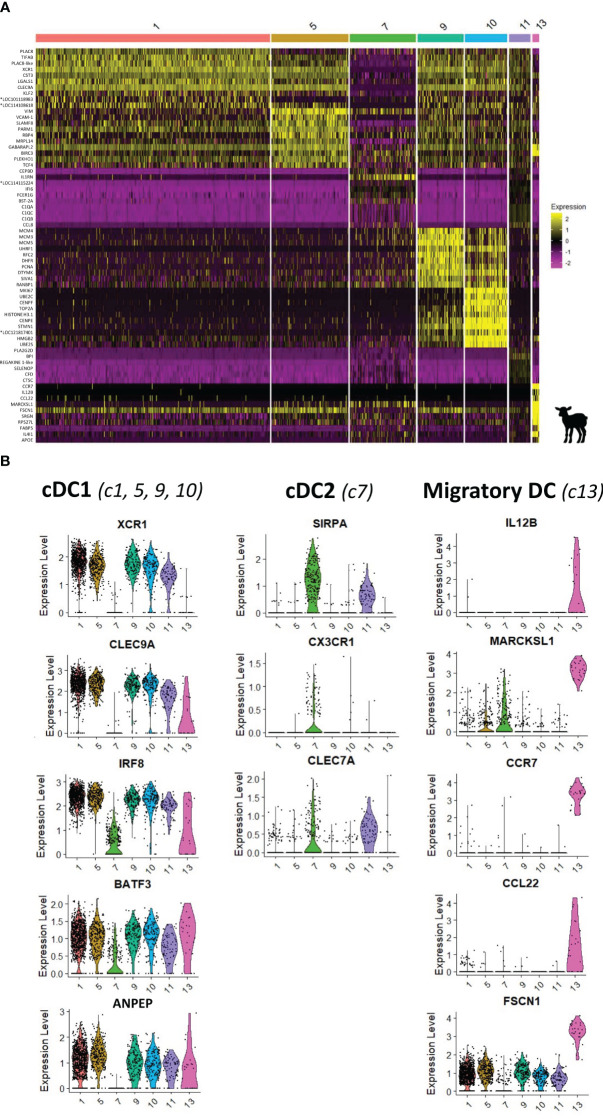
Characterization of dendritic cells from the ileal Peyer’s patch of lamb by scRNA-sequencing. **(A)** Heatmap showing the top 10 differentially expressed genes (p_val_adj) in *FLT3*-expressing clusters (clusters 1, 5, 7, 9, 10, 11, and 13), as determined by Seurat’s FindAllMarkers function. Expression levels are visualized from low expression (pink) to high expression (yellow). Complete gene lists are given in **Supplementary Table 4**. **(B)** UMAP and Violin plots showing the expression of signature genes for type 1 conventional dendritic cells (cDC1) (*XCR1*, *CLEC9A*, *BATF3*, *ANPEP*, *IRF8;* c1, 5, 9, and 10), type 2 cDC (cDC2) (*SIRPA*, *CX3CR1*, and *CLEC7A*; c7), and migratory DC (*IL12B*, *MARCKSL1*, *CCR7*, *CCL22*, and *FSCN1*; c13) for each DC cluster. Expression levels are visualized from low expression (gray) to high expression (blue). *Genes of unknown function.

Through flow cytometry, three subsets of DCs were phenotypically identified and suggested to be cDC1, cDC2, and migratory DCs with targeted gene expression analysis. Based on scRNA-seq data, a heatmap of the top 10 (adjusted *p*-value) DEGs between the seven FLT3-expressing clusters is shown in **Figure 5A** and the complete gene list is provided in **Supplementary Table 4**. As shown in **Figure 5B**, the co-expression of key subset-specific genes clearly confirmed clusters 1, 5, 9, and 10 to be cDC1 (*ANPEP, XCR1, CLEC9A, BATF3*, and *IRF8*). Clusters 9 and 10 encompassed many genes of the cell cycle, with *PCNA, MCM3, MCM4, MCM5*, and *SIVA1* expression that characterizes the S-phase of cells higher in c9 cells compared to cells of c10 expressing predominantly *MKI67, CENPF*, and *TOP2A* that characterize the G1-phase (**Supplementary File 8A, B**). In addition, enriched GO terms were typical for dividing cells such as nuclear division and nuclear DNA replication (**Figure 4E**). As visualized in the UMAP projection (**Figure 4**) and according to specific gene expression, c10 conveys the impression of giving rise to c9 that later diverges to meet non-proliferative cDC1 clusters (c1 and c5). Clusters 1 and 5 shared cDC1 gene signature, but when compared, significant genes including *PLAC8*, *ATP1B1*, and *LGALS1* were highly expressed in c1 compared to c5, which was enriched in transcripts for *MRPL14, SLAMF8, VIM*, and *RBP4* genes, suggesting some diversity among cDC1 cells (**Supplementary File 8C**; **Supplementary Table 6**). Moreover, GO terms enriched for c1 were related to the negative regulation of immune response (e.g., negative regulation of immune system process/defense response/response to external stimulus) while those for c5 referred to immune cell activation (e.g., leukocyte/lymphocyte/T-cell activation involved in immune response) (**Supplementary File 8D**).

On the UMAP dot plot, c7, which expressed *FLT3* gene, is closely localized to the *CSF1R*-expressing clusters corresponding to monocytic cells (**Figure 4A**). Compared to other *FLT3*-expressing clusters, c7 expressed a very low level of cDC1 key genes (*IRF8, CLEC9A, XCR1*, *BATF3*, and *ANPEP*) but a high level of *SIRPA*, *CX3CR1*, and *CLEC7A*, and was therefore designed as cDC2 (**Figure 5B**). Notably, c7 stood out because of its high expression of *IL1RN, IFI6, FCER1G, BST2A*, and *CCL8* (**Figure 5A**).

Cells in c13 showing an isolated projection in the UMAP encompassed specific gene expression of migratory DCs such as *CCR7, IL12B, CCL22, FSCN1, SAMSN1, IDO1, IL4L1*, and *MARCKSL1* (**Figure 5**). This cluster could correspond to the cDC no. 3 cell population previously identified by flow cytometry as CD11c^+^MHCII^+^CD14^−^CD11b^−^Cadm1^−^.

**Figure 6:**
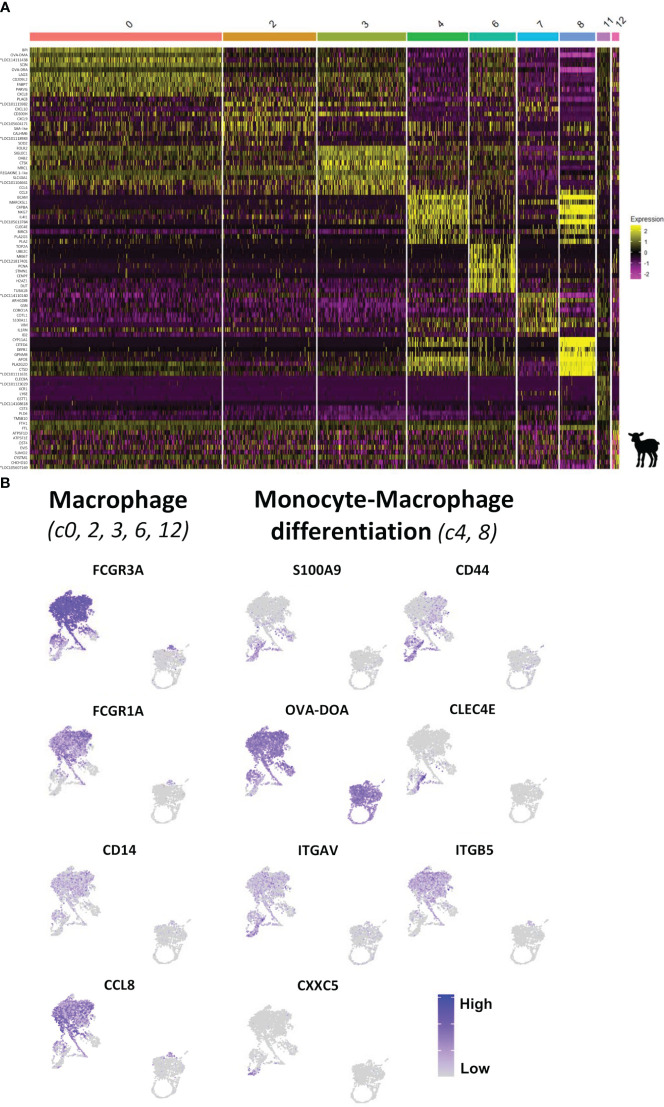
Characterization of monocytic cells from the ileal Peyer’s patch of lamb by scRNA-sequencing. **(A)** Heatmap showing the top 10 differentially expressed genes (p_val_adj) in *CSF1R*-expressing clusters (clusters 0, 2, 3, 4, 6, 7, 8, 11, and 12), as determined by Seurat’s FindAllMarkers function. Expression levels are visualized from low expression (pink) to high expression (yellow). Complete gene lists are given in **Supplementary Table 7**. **(B)** UMAP plots showing the expression of signature genes for macrophages [*FCGR3A* (*CD16*), *FCGR1A* (*CD64*), *CD14*, and *CCL8*; c0, 2, 3, 6, and 12] and of key genes characterizing the intestinal monocyte-to-macrophage differentiation [*S100A9*, *CXXC5*, *CLEC4E*, and *OVA-DOA (MHCII)*; c4 and 8] for each DC cluster. Expression levels are visualized from low expression (gray) to high expression (blue). *Genes of unknown function.

Based on CSF1R expression, monocyte and MAC characterization resulted in the identification of eight clusters with distinct gene expression profiles (**Figure 4A**). Clusters 0, 2, 3, and 6 highly expressed genes commonly associated with MAC such as *FCGR3A (CD16), CCL8, CD14, FCGR1A (CD64), OVA-DRA*, and *OVA-DMA* (**Figure 6**; **Supplementary File 9A**; **Supplementary Table 7**). Among these clusters, c0 can be assigned to the main MAC cluster identified in the bovine MLN by Barut et al. (42) (**Figure 6A**; **Supplementary Table 9**) and c2 and c3 presented pro-inflammatory and anti-inflammatory MAC signatures. Cluster 6 was enriched in cell cycle-related genes including *TOP2A, MKI67, PCNA, CENPF*, and *MCM5* and thus may represent a population of (tissue-resident) proliferating MAC that could participate in the replenishment of *bona fide* intestinal MAC by self-renewal, as reported in other species (17, 34, 43, 44) (**Figure 6**; **Supplementary Files 9D, E**).

A continuous increase in MAC-associated genes such as *FCGR1A*, *OVA-DRA*, *OVA-DMA*, and *C1R* from c8 via c4 toward c0, c2, and c3 (**Figure 6**; **Supplementary Table 8**) was observed. Notably, among the most significant genes with higher expression in c4 compared to c8 were antigen presentation-related genes (*OVA-DRA*, *OVA-DMA*, *OVA-DQA*, and *CD74*) (**Supplementary File 9C**). Cells in c8 and c4 also expressed high levels of *CD44*, *CLEC4E*, and *S100A9*, reported to characterize monocytes and early MAC in the human intestine (17, 34), as well as *VIM*, *S100A11*, and *ALO5XP*, associated with monocytes in the bovine MLN (42) (**Figure 6**; **Supplementary Files 9A, C**). Moreover, c8 exclusively expressed *CXXC5*, a regulator of the differentiation of hematopoietic progenitors toward monocyte development (45) (**Figure 6B**; **Supplementary File 9C**). Finally, expression of αVβ5 integrin, requiring the gene expression of both subunits *IGTAV* and *ITGB5*, was associated with mature MAC in the murine intestine (46). In our dataset, while expression of *ITGAV* was common for CSF1R-expressing clusters, *ITGB5* expression was almost absent from c8 and increased from c4 to c0, c2, and c3 (**Figure 6**). Taken together, these data suggest that a monocyte-to-MAC differentiation path from c8 considered as monocytes recently arrived in the intestinal mucosa, via c4 as an intermediary differentiation stage of MAC, toward mature MAC in c0, c2, and c3.

### 3.5 Study of parasite load and clinical signs of lambs during *Cryptosporidium parvum* infection

**Figure 7:**
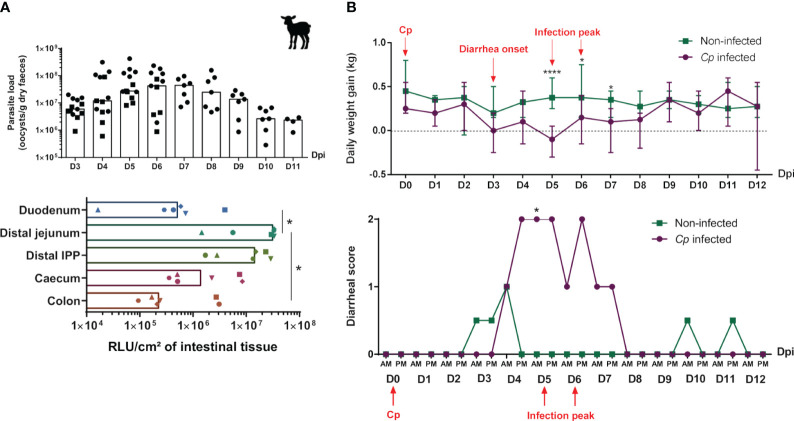
Evolution of parasite load and clinical signs of lambs during *Cryptosporidium parvum* infection. Three-day-old lambs (*n* = 6–7/group/experimentation) were infected or not with 2 × 10^6^ oocysts of *Cryptosporidium parvum* (*Cp*) (INRAE-nluc transgenic strain) by oral route within two independent experimentations [the first ending at 6 days post-infection (dpi) (D6), and the second at 11–12 dpi (D11–12)]. (**A**, top panel) Parasite excretion was monitored daily by counting of oocysts in feces of infected lambs from D3 to D6 (black square) or D11 pi (black circle), and expressed as oocysts per gram of dry feces (median), with each point corresponding to one animal. (**A**, bottom panel) Intestinal parasite load was determined by a measure of luciferase activity of *Cp* transgenic strain at D5–D6 pi in the duodenum, distal jejunum, distal ileal Peyer’s patch (IPP), cecum, and colon of infected lambs, and expressed as relative light unit (RLU) per cm² of intestinal tissue (median), with each dot corresponding to one animal. **(B)** Clinical signs of lambs from the non-infected (green) and *Cp-*infected groups (purple) were monitored from D0 to D12 pi. (**B**, top panel) Daily weight gain (DWG) was calculated as the difference between each day weight and the previous day weight/between two consecutive day (DN − DN−1), and expressed in kilograms (median ± range). DWG at D0 (day of *C parvum* inoculation at 3–4 days of age) corresponds to DWG(D0) − DWG(D−1). DWG > 0 corresponds to weight gain, DWG = 0 corresponds to no change in weight and DWG < 0 corresponds to weight loss. (**B**, bottom panel) Fecal index is represented by a diarrheal score ranging from 0 to 2, with 0 corresponding to normal feces, 1 to semi-liquid feces, and 2 to liquid feces. Statistical analyses were performed using Kruskal–Wallis non-parametric test followed by Dunn’s multiple comparison test to compare the medians of data values between dpi (**A**, left panel), intestinal segments (**A**, right panel), or non-infected and *Cp-*infected groups **(B)**; statistical significance was determined by a *p*-value < 0.05 (*p < 0.05, ****p < 0.0001).

The kinetics of *C. parvum* infection in lambs was evaluated by daily monitoring of parasite excretion in the feces of animals, revealing that the peak of infection occurred at 5–6 dpi (**Figure 7A**), as previously observed in lambs with other *C. parvum* strains (47). The fecal parasite load then gradually decreased over time, leading to an approximate 100-fold decrease at 10 dpi, and thereafter, parasites started to become undetectable in the feces of lambs. In addition, the parasite load in different intestinal segments of lambs was measured with the N-Luc signal at the peak of infection and revealed the highest level of infection in the distal jejunum and IPP compared to the duodenum and colon (approximately 100 times less) (**Figure 7A**), confirming the preferential tropism of *C. parvum* for the distal part of the small intestine in this species. Clinical signs of lambs including body weight gain and fecal index were also daily monitored. Compared to the linear growth of uninfected animals, the weight of infected lambs stagnated from the onset of diarrhea (3 dpi) and decreased significantly at the peak of infection (5 dpi) (**Figure 7B**). Their daily body weight gain became similar to that of uninfected animals only after the end of the diarrheal episode (8 dpi).

### 3.6 Intestinal immune responses of lambs during *Cryptosporidium parvum* infection

**Figure 8:**
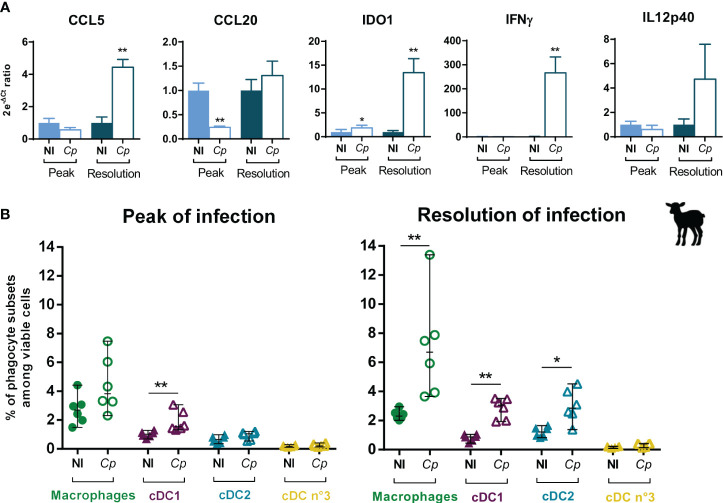
Immune responses and mononuclear phagocyte recruitment/proportion in the ileal Peyer’s patch of lambs during *Cryptosporidium parvum* infection. Three-day-old lambs were infected or not (NI) with 2 × 10^6^ oocysts of *Cryptosporidium parvum* (*Cp*) by oral route within two independent experimentations (*n* = 6–7/group/experimentation) and their ileal Peyer’s patches (IPP) were sampled at 6 days post-infection (dpi) (peak of infection) or 11–12 dpi (resolution of infection). **(A)** IPP were processed for total RNA extraction, and mRNA expression of CCL5, CCL20, IFNγ, IL12p40, and IDO1 genes was analyzed by RT-qPCR with the FLUIDIGM method. Data are expressed as the ratio between the 2e^-ΔCt^ values of individuals and the mean of 2e^-ΔCt^ values of non-infected lambs (mean), following normalization with three reference genes (*HPRT*, *GAPDH*, and *ACTB*) (mean ± SEM). **(B)** Proportions of macrophages (green), cDC1 (purple), cDC2 (blue) and cDC no. 3 (yellow) in the IPP were analyzed by flow cytometry following mechanical and enzymatic dissociations of intestinal tissues, isolation of total isolated intestinal cells, and staining. Data are expressed in percentage among viable cells (median ± range) with each dot corresponding to one animal. NI, non-infected. Statistical analyses were performed using Mann–Whitney non-parametric test to compare the medians of gene expression and proportions of mononuclear phagocyte subsets between infected and non-infected animals; statistical significance was determined by a *p*-value < 0.05 (*p < 0.05, **p < 0.01).

To better understand the local immune responses to *C. parvum* infection in the intestine of ruminants, we analyzed the gene expression of known effectors of the anti-*Cryptosporidium* immune response in the intestine of infected lambs at two key time points: the infection peak, during which the innate immune response starts to control the parasite multiplication, and at 11–12 dpi, when the last parasites are very efficiently eliminated. The infection induced an increase of the gene expression of the chemokine CCL5 and the cytokine IFNγ (approximately 250 times) in the IPP of lambs during the resolution of infection (**Figure 8A**), which we also observed in the distal jejunum and JPP (**Supplementary File 10**). We also observed an upregulation of the *IL12p40* cytokine gene expression in the intestinal segments, but it was not significant (**Figure 8A**; **Supplementary File 10**).

Moreover, the infection induced the increase of the *IDO1* gene expression in the distal small intestine of lambs from the peak of infection (**Figure 8A**; **Supplementary File 10**). IDO1 is an enzyme capable of catabolizing the tryptophan necessary for the parasite development, and is notably produced following the increase of the transcriptional factor STAT-1 in intestinal epithelial cells, induced by IFNγ (48). Thus, the weak increase of *IDO1* in IPP at the peak of infection that subsequently intensified at 11–12 dpi could be linked to the increase of *IFNγ* observed only during the resolution of infection (**Figure 8A**).

Finally, results showed a decrease of the *CCL20* gene expression at the peak of infection but no longer during the resolution (**Figure 8A**; **Supplementary File 10**). Similar results were observed in the neonatal murine model of *C. parvum* infection, with the highest diminution of *CCL20* in the intestine at the peak of infection, but maintained at a lower amplitude in the later stages of the infection (49).

### 3.7 Evaluation of mononuclear phagocyte proportions in the intestine of lambs and calves during *Cryptosporidium parvum* infection

DCs, and especially cDC1, play a key role in controlling the acute phase of *C. parvum* infection in the murine neonatal model (7, 8, 50). Thus, to better characterize the intestinal cellular immune response to *C. parvum* infection in ruminants, we analyzed the abundance of MP subsets in the intestine of lambs and calves during the infection by flow cytometry. During the phase of parasite control (11–12 dpi), data highlighted an increase in MAC and cDC subset proportions except for the minor cDC no. 3 subset, with notably an earlier upregulation of cDC1 proportion already visible from the peak of infection (5 dpi) (**Figure 8B**; **Supplementary File 11**). In general, these results were observed regardless of the location in the intestine (ileum or jejunum) and the type of tissue (lymphoid or not). The increase of these cell subsets could be attributed to cell recruitment from blood precursors or to *in situ* proliferation.

The increase of cDC1 and MAC proportions in all three intestinal tissues in response to *C. parvum* infection was also found in calves at 11–12 dpi during the resolution of infection, as well as the lack of variation for cDC no. 3, but in contrast to the cDC2 proportion, which was only slightly increased in the IPP (**Supplementary File 12**).

## 4 Discussion

Mononuclear phagocytes are key players in intestinal tissue homeostasis, which relies on a fine balance between immune tolerance and appropriate local immune responses, based on their ability to distinguish between harmless and pathogenic antigens (51). Intestinal cDCs play a major role in inducing oral tolerance to food and commensal antigens (52) and in promoting intestinal IgA production, essential for mucosal barrier protection (53). As professional APCs, cDCs continuously sample the intestinal microenvironment with specific mechanisms for the uptake of luminal antigens, such as transepithelial dendrite protrusions (54, 55) and antigen transfer from tissue-resident MAC (TRM) through gap junctions (56) or from Goblet and M cells (57, 58). Complementarily, MAC are efficient scavengers specialized in clearing of microbes and apoptotic cells, tissue repair, and remodeling, and contribute to maintain oral tolerance (43, 51, 59, 60). In human and mouse, the intestinal MP system harbors a wide phenotypic and functional diversity of cDC and MAC subsets depending on the intestinal segment, the lymphoid or non-lymphoid composition of the tissue, and their location within the tissue (16, 17, 44, 61–64). Moreover, neonates display a distinct intestinal immune system that fundamentally differs from adults, with innate and adaptive immune components undergoing a constant process of differentiation and adaptation, and impaired efficiency in their microbial response (65).

MPs are far less described in ruminants. In the present study, owing to phenotypic and targeted gene expression analyses, we identified one population of MAC and three subsets of cDC (cDC1, cDC2, and a minor cDC subset with migratory properties) in lymphoid and non-lymphoid tissues in lamb and calf distal small intestine. Unsupervised single-cell transcriptomic analysis confirmed the identification of these four MP subpopulations in the IPP of lamb, with a major cDC1 subset among cDCs, while highlighting a deeper diversity of cell states or subsets among monocytic and DCs.

A striking observation is the rapid postnatal reversal between the cDC1 and cDC2 proportions in the small intestine of lambs, with cDC1 being under-represented compared to cDC2 at birth and becoming strongly dominant from 10 days old (**Figure 2A**; **Supplementary File 4**). Age-dependent differences in the intestinal immune system have already been observed in lamb and goat kid models, such as stronger cytokine response to TLR stimulation by neonatal MLN cells compared to older animals (66, 67) or greater proportion of CD14^+^CD11b^+^CD40^+^ cells in neonatal than adult MLN cells (67). Recently, Torow et al. also showed age-dependent changes in the mouse PP MP subset composition with cDC1 enriched in neonatal PP while cDC2 were reduced in comparison to the adult host (68). From birth, the progressive colonization of the intestine by microbes plays an important role in educating the intestinal immune system (65). Hence, the early change in cDC proportions observed could be linked to the installation of the intestinal microbiota (69–71). In fact, there are significant differences in the ratio of cDC1 to cDC2 along the murine and human intestine, with cDC1 enriched in the PP and in the colon in comparison to the upper intestine (32, 51, 63, 72), potentially related to the presence of distinct microbiota (73). Notably, Moreira et al. recently highlighted a regional-specific compartmentalization of distinct murine intestinal DC signatures with a differential distribution throughout the small intestine and colon, probably resulting from different exposures to foreign antigens, as these are predominantly dietary in the small intestine and derived from the microbiota in the large intestine, and therefore from different immune responses (63). Furthermore, in a germ-free mouse model, microbiota-derived signals were shown to promote cDC2 and RORγt^+^ APC maturation in PP after birth, thereby influencing the cDC subset composition (68). Overall, our data support the observation that the distribution of cDC subsets in the intestine is tissue-, age-, and species-specific.

Another surprising feature of our data is the identification of cDC1 as the major DC subset in the small intestine of lambs and calves from 10 days after birth, in both lymphoid and non-lymphoid tissues. Indeed, across species, cDC2 are usually described as the dominant cDC subset in various tissues, including the intestine (72, 74, 75). Notably, in neonatal and adult mice, the major CD103^+^ subpopulation of cDC2 (CD11b^+^CD172α^+^) is the most abundant in the small intestine, in comparison to the cDC1 subset (CD103^+^CD11b^−^XCR1^+^) which is approximately two to three times less represented (8, 38, 63, 74, 76). Likewise, Granot et al. have demonstrated that cDC2 (CD172α^+^CD1c^+^) is the predominant cDC subset in the intestine (jejunum and PP) of young children (0–9 years old) and adults (72).

In the present study, we did not observe marked differences between MP subsets populating PP and non-lymphoid tissues in the intestine of lambs and calves, in contrast to the murine model (64). In terms of PP structure, it is possible to isolate domes in mice with a limited number of dome-associated villi (DAV) from villi for tissue digestion, whereas in ruminants, PPs are a long continuum of domes, DAV, and villi. As a result, the MP population we have isolated from PP is a mixture including both dome and lamina propria cells.

This study is the first fine characterization of the MP system in the PP of young ruminants. Earlier works started to describe the phenotype and distribution of MP subpopulations in the IPP of calves and lambs (77, 78). However, based on the analysis of the co-expression of a limited number of markers, these studies may not clearly discriminate between subsets and therefore hardly reflect the current heterogeneity of the MP system. For instance, Fries et al. demonstrated that diverse myeloid subpopulations and significant differences in regional distribution are established early in life in the calf intestine (78). They suggested a potentially higher proportion of DCs (CD11c^hi^ cells co-expressing either CD26 or CD205) than MAC (CD11c^hi^ cells co-expressing either CD14, CD11b, or CD172α), whereas the CD11b and CD172α markers are now known to be also associated with cDC2.

Our scRNA-seq analysis identified a majority of monocytic cell and cDC1 clusters and minor cDC2 and CCR7-expressing activated DC clusters in the lamb IPP (**Figure 4**). In parallel, Torow et al. recently showed that the neonatal PP in mice harbors a reduced monocytic cell proportion compared to DCs, the identification of a RORγt^+^ DC subset, and the distinct clustering of activated cDC1 and cDC2 from their corresponding quiescent cDC1 and cDC2 counterparts (68). Given that PP development and maturation differ between ruminants and mice (79), it is not surprising to observe species-specific differences of MP subset composition in neonates.

Although sharing phenotypic and functional properties, the cDC system of mouse PP is enriched with distinct populations of DC and MAC in comparison to those of the lamina propria, finely characterized by Lelouard’s team (38, 64, 80, 81). Notably, a hallmark of the SED of PP is the existence of specialized lysozyme-expressing DCs derived from monocytes, termed LysoDC, representing the main phagocyte subset together with MAC that share lysozyme expression (80). In addition, a population of cells phenotypically related to LysoDCs were shown to populate human PP (81). Based on our phenotypic characterization of MP subsets in the IPP of lambs and calves (**Figure 1**; **Supplementary File 3**), and according to the surface marker expression of LysoDC in mice, such a population would be part of the CD172α^+^CD11b^+^ population that we identified as either cDC2 or MAC. However, lysozyme gene expression was detected in all four MP subsets that we discriminated by flow cytometry, with the highest level in cDC1 (**Figure 3B**), as well as in all DC and MAC clusters in our scRNA-seq analysis (data not shown). Overall, murine LysoDCs display features of monocyte-derived cells but differ from MAC by a gene signature related to DC functions (80). In our scRNA-seq analysis, cluster 13 and part of cluster 7 cells showed mixed expression of both MAC and DC signatures, suggesting the presence of monocyte-derived DC in the IPP of lamb, as described in human and mouse PPs.

Intestinal cDCs contribute to the protective immune responses against pathogens, such as cDC1, which are key players in controlling the early phase of infection by the parasites *Toxoplasma gondii* (82), *Leishmania major* (83), and *C. parvum* (8). In the mouse model, our laboratory correlated the low proportion of CD103^+^ DC in the intestine of newborns until weaning with the increased sensibility to *C. parvum* infection (7), notably in association with the crucial role of intestinal CD103^+^CD11b^−^ DC (Batf3-dependent) (cDC1) in the early control of the parasite (8). In contrast to rodent models, depletion of specific cell subpopulations in large animal models such as lambs faces major technical constraints, which considerably limit functional studies. However, our data support the idea that intestinal cDC1 should be important effectors in the protection against *C. parvum* in ruminants. Indeed, the weak proportion of cDC1 observed in the intestine of newborn lambs during the first 10 days (**Figure 2**) is correlated with their high sensibility to *C. parvum* infection, as observed in mice (7). In addition, the increase of intestinal cDC1 in response to the infection from the peak of infection (**Figure 8B**) may indicate their importance for efficient parasite control.

The kinetics of the intestinal immune response in the neonatal murine model of *C. parvum* infection, described in detail by our laboratory, encompass the production of a wide range of chemokines (CCL5, CXCL10, CXCL9, etc.) and cytokines (IFNγ, IL12, IL18, etc.) favoring recruitment and activation of diverse immune cell types (7, 84–87). The immune response we observed in the lamb IPP (**Figure 8A**), one of the most infected intestinal tissues (**Figure 7A**), is consistent with the IFNγ, IL12p40, and CCL5 responses described in the ileum of infected neonatal mice (84, 86) and what we previously observed in young lambs even if the kinetics of response differed most probably due to the difference of age at the day of the inoculation (47).

*C. parvum* has developed strategies to escape host protection by subverting some innate immune effectors such as CCL20 and IDO1 (48, 49). The CCL20 chemokine, in addition to its known chemotactic activity toward CCR6+-immune cells, was described to display antimicrobial activity against the *C. parvum* parasite. As CCL20 production was reduced in the ileum and the intestinal epithelial cells of neonatal mice during *C. parvum* infection, a strategy of immune evasion induced by the parasite has been suggested. In the intestine of lambs, CCL20 expression is also significantly reduced at the peak of infection (**Figure 8**) and corroborates the observation in the mouse model of infection. Moreover, *C. parvum* is able to reduce the STAT1-induced IDO1 expression in mouse intestinal epithelial cells, thus restoring tryptophan availability to allow parasite development (48), consistent with the weak increase of IDO1 in the IPP of lamb at the peak of infection compared to 11–12 dpi when parasites are efficiently cleared (**Figure 8**). Hence, our results suggest the existence of some escape mechanisms in young ruminants as previously observed in neonatal mice. The ability of *C. parvum* to circumvent immune response may take variable forms in neonates between animal species. For example, young infants are still highly susceptible to cryptosporidiosis at least until 5 years while young ruminants and mice become strongly resistant to the infections in only a few weeks.

In this work, we described in detail the different MP populations and subsets in the intestine of young ruminants and their mobilization and response during intestinal infection by *C. parvum*. These results are insightful to design strategies for modulating the immune response and boost immunity in very young animals and better understand host–pathogen interactions.

## Data availability statement

The data presented in the study are deposited in the ENA repository, accession number PRJEB71947 (https://www.ebi.ac.uk/ena/browser/view/PRJEB71947).

## Ethics statement

The animal study was approved by Val de Loire Ethics Committee, France (CEEA19) (no. APAFlS#21604-201907250902391 v2 and #21515-2019071714558143 v2). The study was conducted in accordance with the local legislation and institutional requirements.

## Author contributions

AB: Formal analysis, Investigation, Writing – original draft, Writing – review & editing. FT: Formal analysis, Writing – review & editing. TC: Methodology, Writing – original draft. CB: Methodology, Writing – review & editing. YL: Methodology, Writing – review & editing. AS: Methodology, Writing – review & editing. TP: Methodology, Writing – original draft. JS: Funding acquisition, Supervision, Writing – review & editing. PP-P: Funding acquisition, Writing – review & editing. FL: Conceptualization, Formal analysis, Funding acquisition, Supervision, Writing – original draft, Writing – review & editing. SL-L: Conceptualization, Formal analysis, Funding acquisition, Investigation, Supervision, Writing – original draft, Writing – review & editing.
