# Supplementary material for: Characterization of intestinal mononuclear phagocyte subsets in young ruminants at homeostasis and during Cryptosporidium parvum infection
Source: Front Immunol. 2024 May 2;15:1379798. doi: 10.3389/fimmu.2024.1379798 (PMC11096452; doi:10.3389/fimmu.2024.1379798)
Supplement: Supplementary file 1 [file DataSheet_1.docx]

Supplementary Material

**Characterization of intestinal mononuclear phagocyte subsets in young ruminants at homeostasis and during *Cryptosporidium parvum* infection**

**Baillou^1,3^, F. Tomal^1^, T. Chaumeil^2^, C. Barc^2^, Y. Levern^1^, A. Sausset^1^, T. Pezier^1^, J. Schulthess^3^, P. Peltier-Pain^3^, F. Laurent^1 *^ and S. Lacroix-Lamandé^1*^**

^1^UMR1282 Infectiologie et Santé Publique, INRAE Centre Val de Loire, Université François Rabelais de Tours, 37380 Nouzilly, France

^2^UE1277 Plateforme d’Infectiologie Expérimentale (PFIE), INRAE Centre Val de Loire, 37380 Nouzilly, France

^3^Phileo by Lesaffre, 137 rue Gabriel Péri, 59700, Marcq-en-Barœul, France.

*These authors share senior authorship

*** Correspondence:** Sonia Lacroix-Lamandé ([sonia.lamande@inrae.fr](mailto:sonia.lamande@inrae.fr)) and Fabrice Laurent ([fabrice.laurent@inrae.fr](mailto:fabrice.laurent@inrae.fr))

# Supplementary Data

Supplementary Material should be uploaded separately on submission. Please include any supplementary data, figures and/or tables.

Supplementary material is not typeset so please ensure that all information is clearly presented, the appropriate caption is included in the file and not in the manuscript, and that the style conforms to the rest of the article.

# Supplementary Figures and Tables

For more information on Supplementary Material and for details on the different file types accepted, please see [here](https://www.frontiersin.org/guidelines/author-guidelines#supplementary-material).

## Supplementary Figures


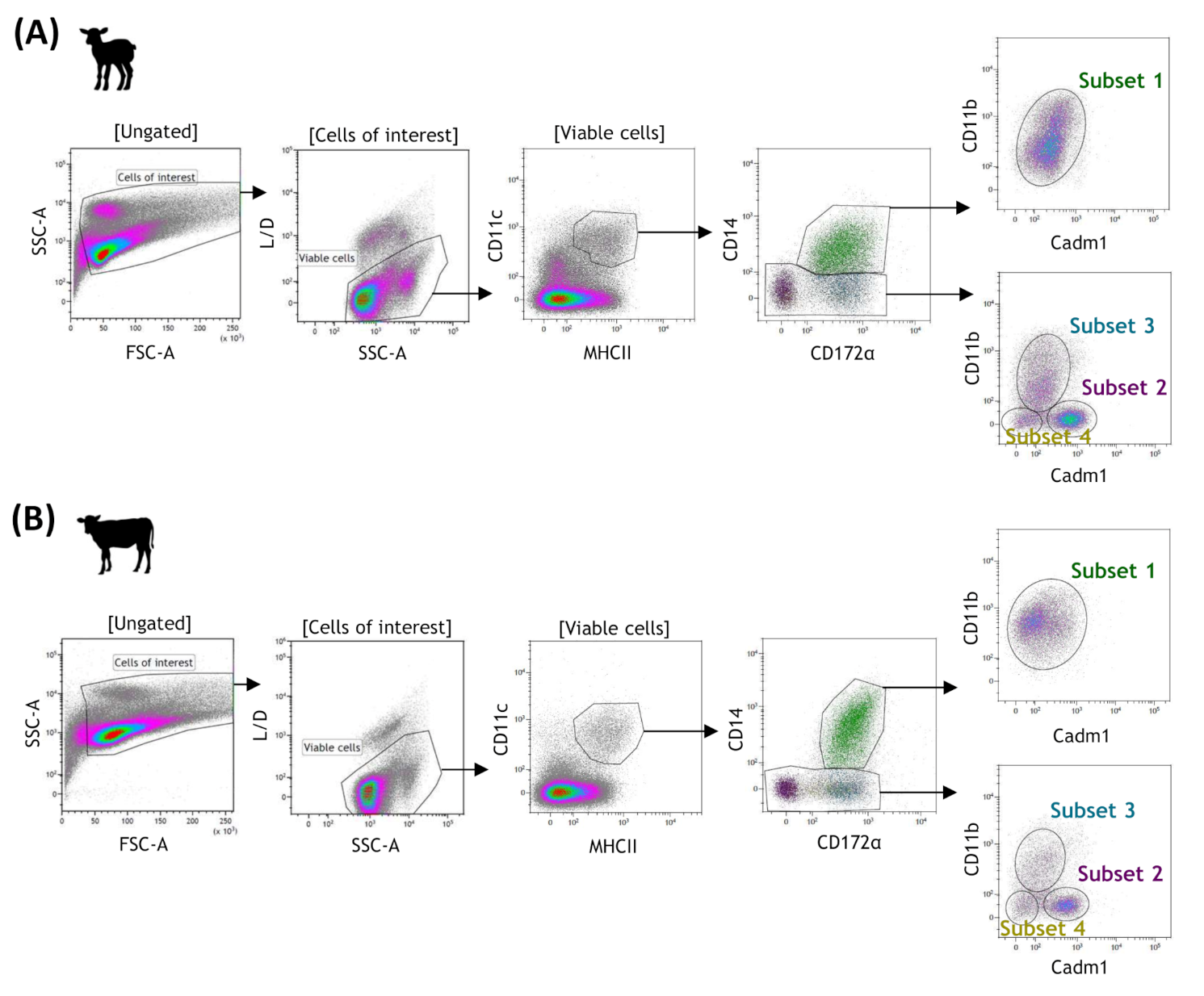


**Supplementary File 1. Flow cytometry gating strategy for the identification of mononuclear phagocytes in the intestine of lambs and calves.** Intestinal tissues from the ileal Peyer’s patch were recovered from a 10-day-old lamb and calf. Mechanical and enzymatic dissociations were performed to obtain total isolated intestinal cells, then stained for flow cytometry. Representative flow cytometry gating strategy for mononuclear phagocytes (MP) subsets is shown for the intestine of lamb (**A**) and calf (**B**). Gating on forward scatter (FSC) and side scatter (SSC) parameters was first used to select cells of interest by exclusion of cell debris (*i.e.* FSC^low^ and SSC^low^ events) and viable cells were next selected by exclusion of dead cells (*i.e.* viability marker (L/D)-positive cells). Then, CD11c^+^MHCII^+^ cells were gated to select total MP and four subsets were distinguished based on surface expression of CD14, CD172α, CD11b and Cadm1, as follows: CD14^+^CD172α^+^Cadm1^int^CD11b^+^ (subset 1, green), CD14^-^CD172α^-^Cadm1^+^CD11b^-^ (subset 2, purple), CD14^-^CD172α^+^Cadm1^int^CD11b^+^ (subset 3, blue) and CD14^-^CD172α^+/-^Cadm1^-^CD11b^-^ (subset 4, yellow).

**
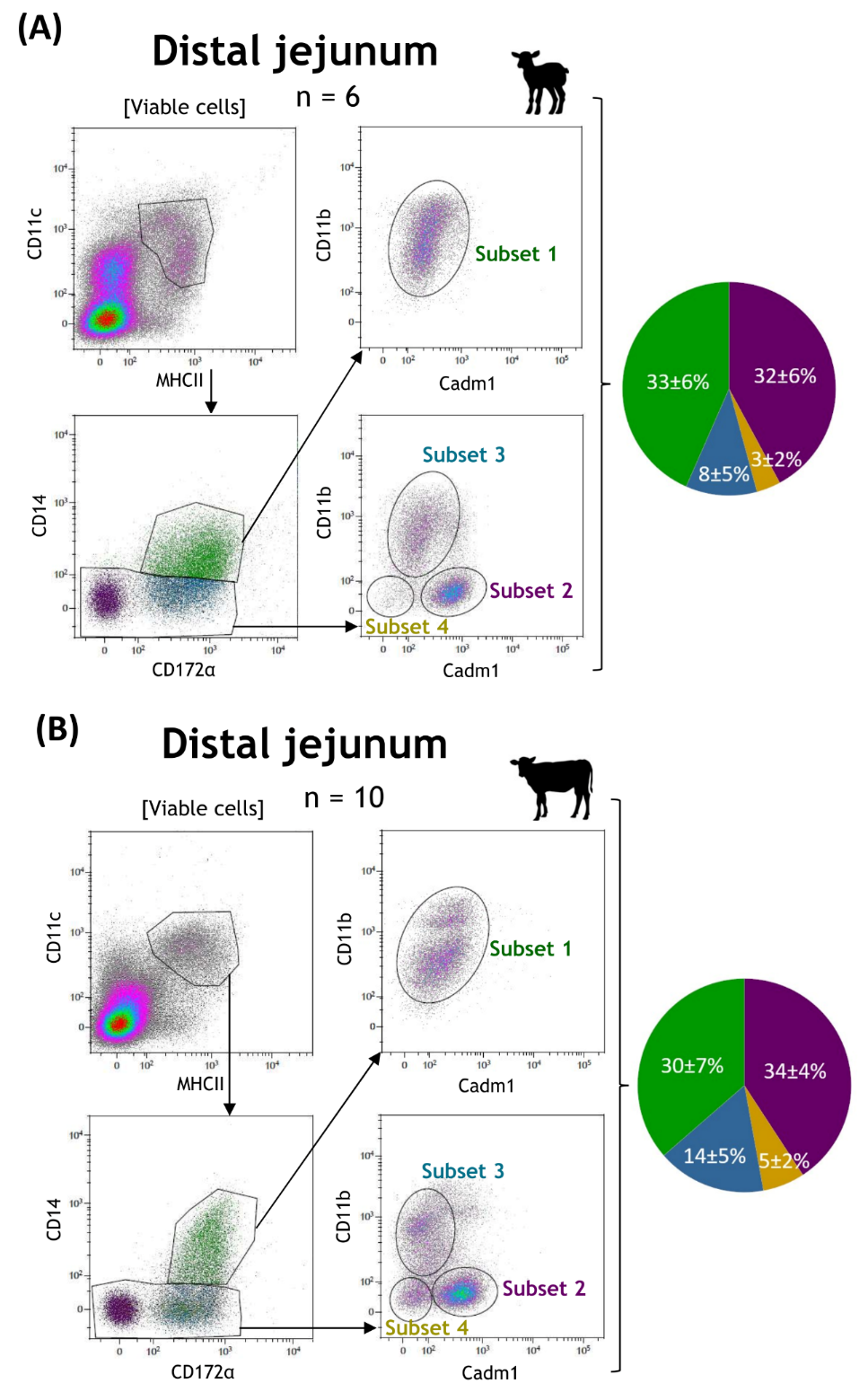
**

**Supplementary Figure 2. Identification of neonatal intestinal mononuclear phagocytes in the distal jejunum of lambs and calves.** Intestinal tissues from the distal jejunum were recovered from 10-day-old lambs (n = 6) and calves (n = 10). Mechanical and enzymatic dissociations were performed to obtain total isolated intestinal cells, then stained for flow cytometry. Representative gating for mononuclear phagocytes (MP) subsets are shown for the IPP of lamb **(A)** and calf **(B)**. Following exclusion of dead cells, CD11c^+^MHCII^+^ cells were gated to select total MP and then, four subsets were distinguished based on surface expression of CD14, CD172α, CD11b and Cadm1, as follows: CD14^+^CD172α^+^Cadm1^int^CD11b^+^ (subset 1, green), CD14^-^CD172α^-^Cadm1^+^CD11b^-^ (subset 2, purple), CD14^-^CD172α^+^Cadm1^int^CD11b^+^ (subset 3, blue) and CD14^-^CD172α^+/-^Cadm1^-^CD11b^-^ (subset 4, yellow). Pie charts represent the cell proportions in percentage of each intestinal MP subset among viable CD11c^+^MHCII^+^ cells (mean ± SD) for lambs (n = 6; **A,** **right panel**) and calves (n = 10; **B,** **right panel**).


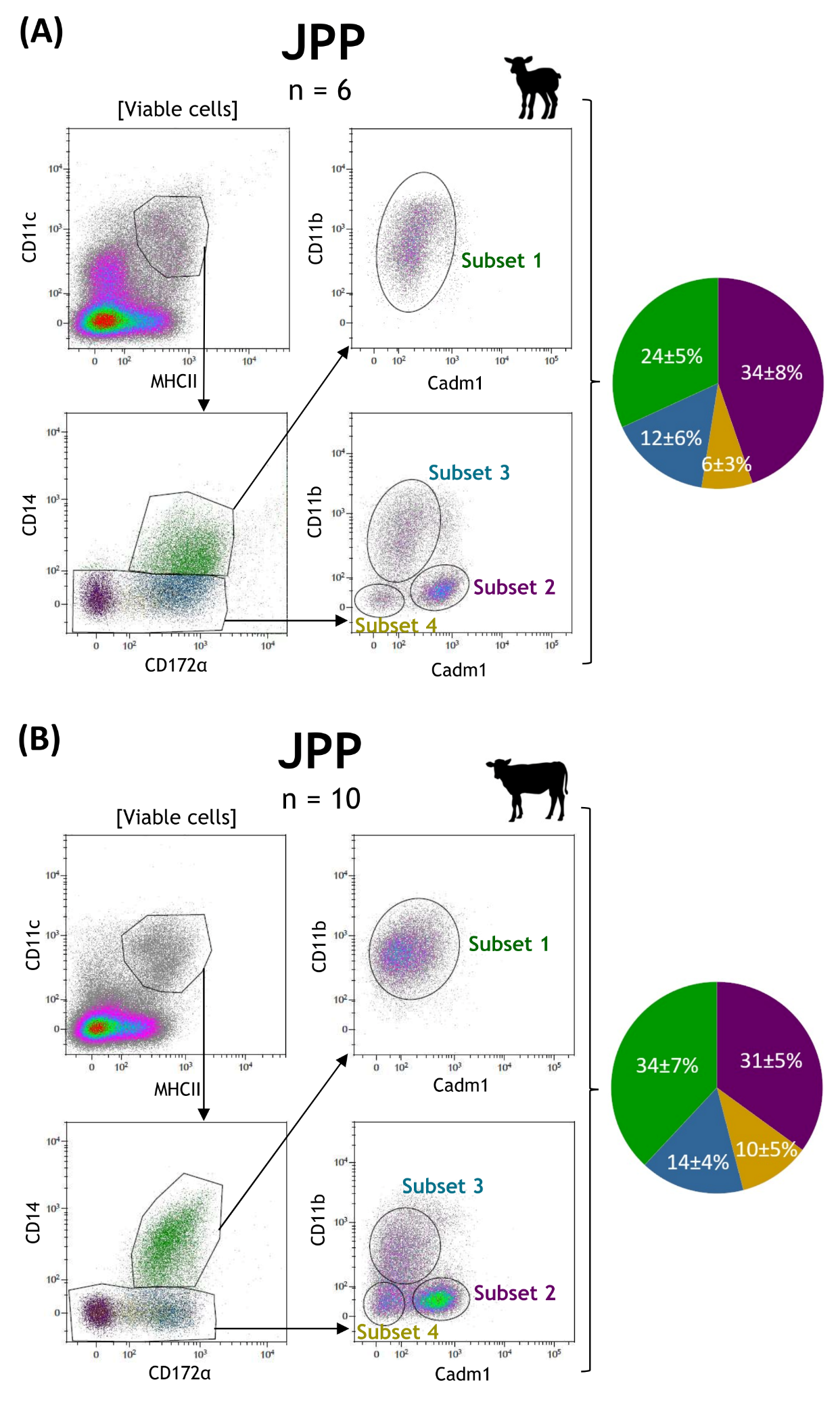


**Supplementary File 3. Identification of neonatal intestinal mononuclear phagocytes in the jejunal Peyer’s patches of lambs and calves**. Intestinal tissues from the jejunal Peyer’s patches (JPP) were recovered from 10-day-old lambs (n = 6) and calves (n = 10). Mechanical and enzymatic dissociations were performed to obtain total isolated intestinal cells, then stained for flow cytometry. Representative gating for mononuclear phagocytes (MP) subsets are shown for the JPP of lamb **(A)** and calf **(B)**. Following exclusion of dead cells, CD11c^+^MHCII^+^ cells were gated to select total MP and then, four subsets were distinguished based on surface expression of CD14, CD172α, CD11b and Cadm1, as follows: CD14^+^CD172α^+^Cadm1intCD11b^+^ (subset 1, green), CD14^-^CD172α^-^Cadm1^+^CD11b^-^ (subset 2, purple), CD14^-^CD172α^+^Cadm1^int^CD11b^+^ (subset 3, blue) and CD14^-^CD172α^+/-^Cadm1^-^CD11b^-^ (subset 4, yellow). Pie charts represent the cell proportions in percentage ofeach intestinal MP subset among viable CD11c^+^MHCII^+^ cells (mean ± SD) for lambs (n = 6; **A, right panel**) and calves (n = 10; **B, right panel**).


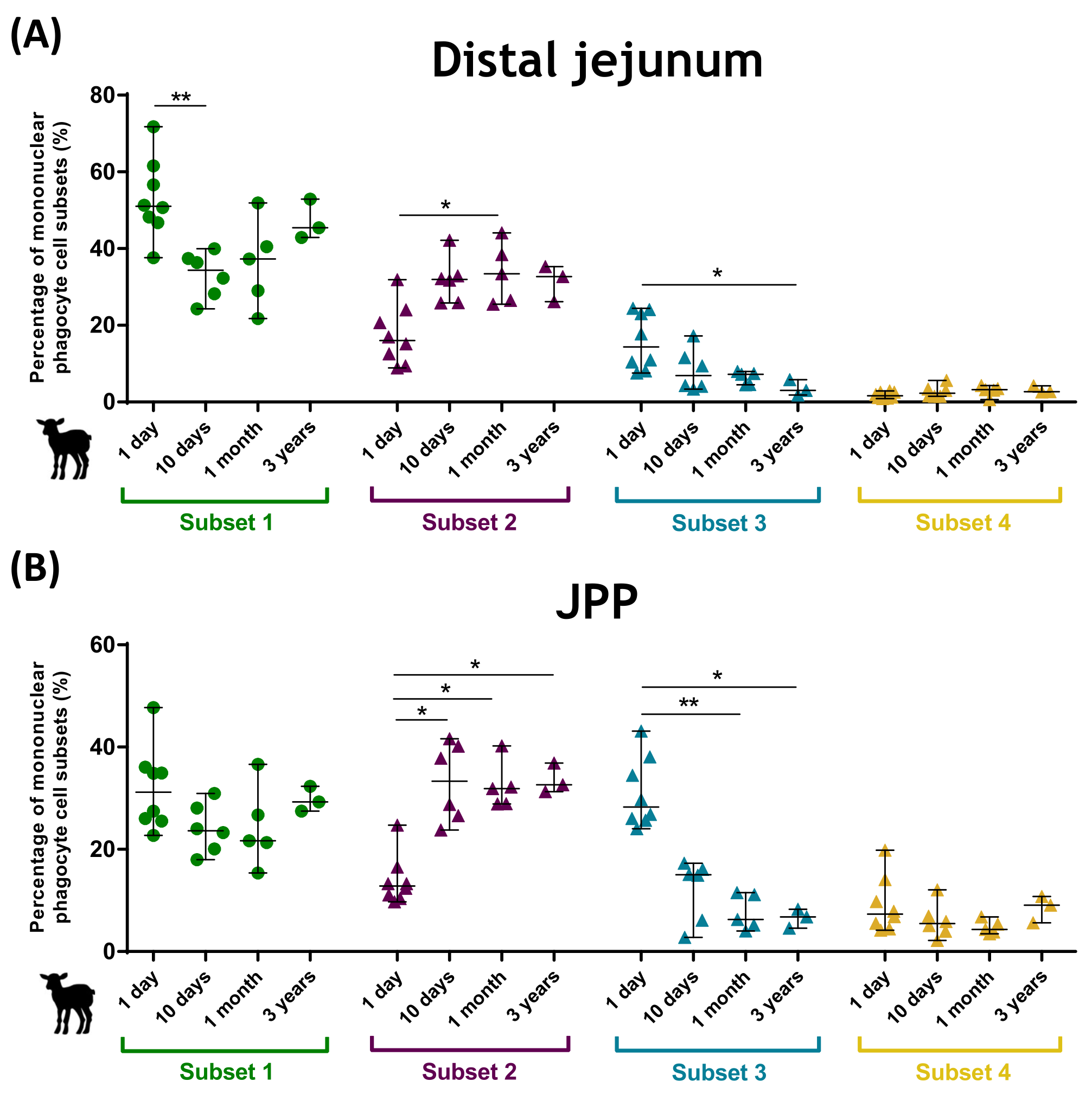


**Supplementary File 4. Age-evolution in the subset proportions of distal jejunal mononuclear phagocytes in lambs.** Intestinal tissues from the distal jejunum were recovered from day-old (n = 8), 10-day-old (n = 6), month-old lambs (n = 5) and from 3-year-old ewes (n= 3). Mechanical and enzymatic dissociations were performed to obtain total isolated intestinal cells, then stained for flow cytometry. Following exclusion of dead cells, CD11c^+^MHCII^+^ cells were gated to select mononuclear phagocytes (MP) and then, four subsets were distinguished based on surface expression of CD14, CD172α, CD11b and Cadm1, as follows: CD14^+^CD172α^+^Cadm1^int^CD11b^+^ (subset 1, green), CD14^-^CD172α^-^Cadm1^+^CD11b^-^ (subset 2, purple), CD14^-^CD172α^+^Cadm1^int^CD11b^+^ (subset 3, blue) and CD14^-^CD172α^+/-^Cadm1^-^CD11b^-^ (subset 4, yellow). Evolution of MP subset cell proportions in distal jejunum (non-lymphoid) **(A)** and jejunal Peyer’s patches **(B)** according to the age of animals, expressed in percentage of total MP (median ± range) with each point corresponding to one animal. Statistical analyses were performed out by Kruskal-Wallis non-parametric test followed by Dunn’s multiple comparison test to compare the medians of MP subsets between age groups; statistical significance was determined by a *P*-value<0.05 (**P*<0.05, ***P*<0.01).


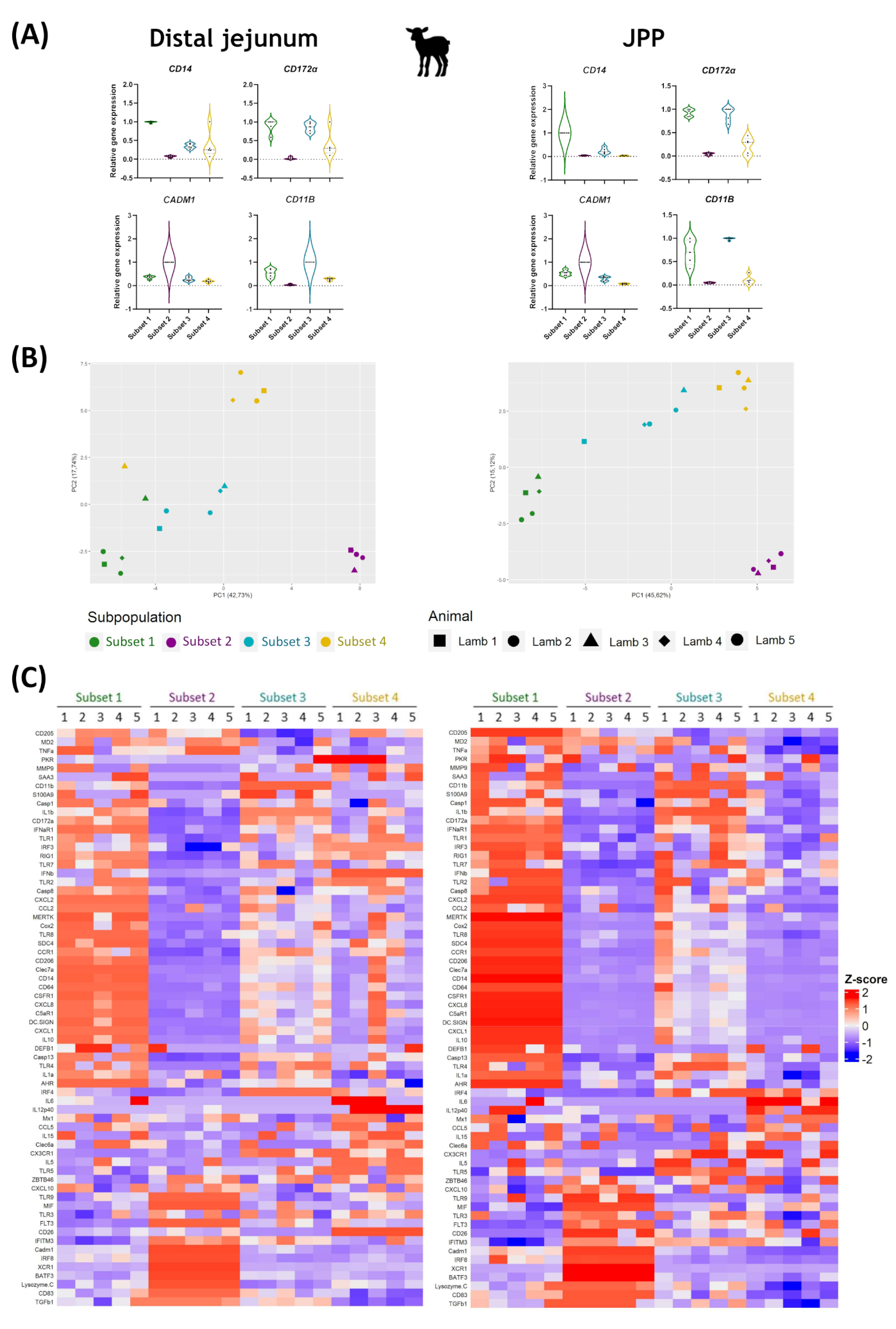


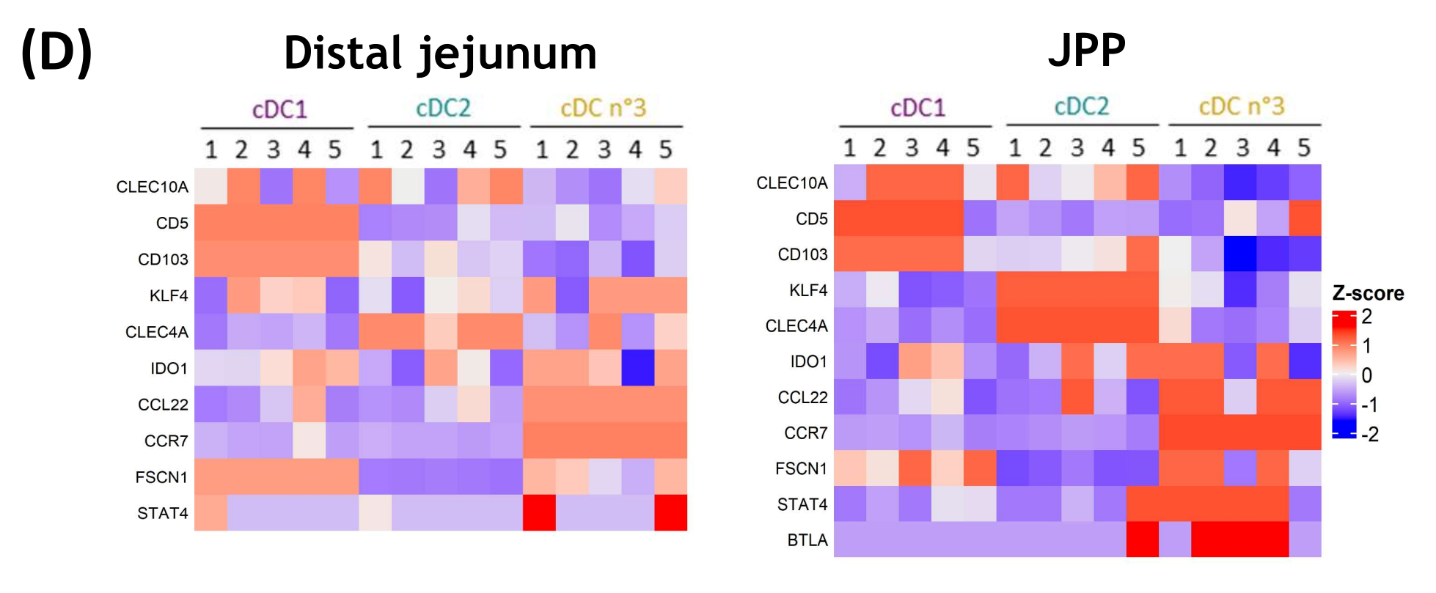


**Supplementary File 5. Transcriptomic analyses of mononuclear phagocyte subsets in the distal jejunum and jejunal Peyer’s patches of lamb.** Gene expression in the four sorted mononuclear phagocyte (MP) subsets from the distal jejunum (**left side**) and the jejunal Peyer’s patch (JPP) (**right side**) of 10-day-old lambs (n = 5) was assessed by classical quantitative RT-PCR **(D)** or with the FLUIDIGM® method **(A, B, C)**. Complete list of the genes analyzed and corresponding primers is provided as **Supplementary Table 1**. Gene expression was defined by relative gene expression levels normalized to maximal expression across cell subsets, following normalization with three housekeeping genes (*HPRT*, *GAPDH*, *ACTB*) and 2e-ΔCt value calculation. (**A**) Expression of the genes coding for the proteins used for cell sorting (*CD14*, *CD172α*, *CADM1*, *CD11B*) in each cell subset, represented by violin plots (median and quartiles). Each point corresponds to one animal. **(B)** Principal component analysis (PCA) represented by its first two dimensions with corresponding variances in percentage and performed on relative gene expression values. The 66 genes displayed in **(C)** were included in the PCA. Each point corresponds to data of one animal, with one point form per individual animal. **(C)** MP subset-specific gene transcription represented by heatmaps of the Z-score normalized relative gene expression values for each gene analyzed in the four MP subsets, with hierarchical clustering of genes. According to their gene expression profile, subset 1 was identified as macrophages (MAC), subset 2 as type 1 conventional dendritic cells (cDC1), subset 3 as type 2 cDC (cDC2) and subset 4 was classified as cDC n°3. **(D)** Transcription of a selected set of genes characterizing DC subsets represented by heatmap of the Z-score normalized relative gene expression values for each gene analyzed in the three DC subsets, with hierarchical clustering of genes.

**
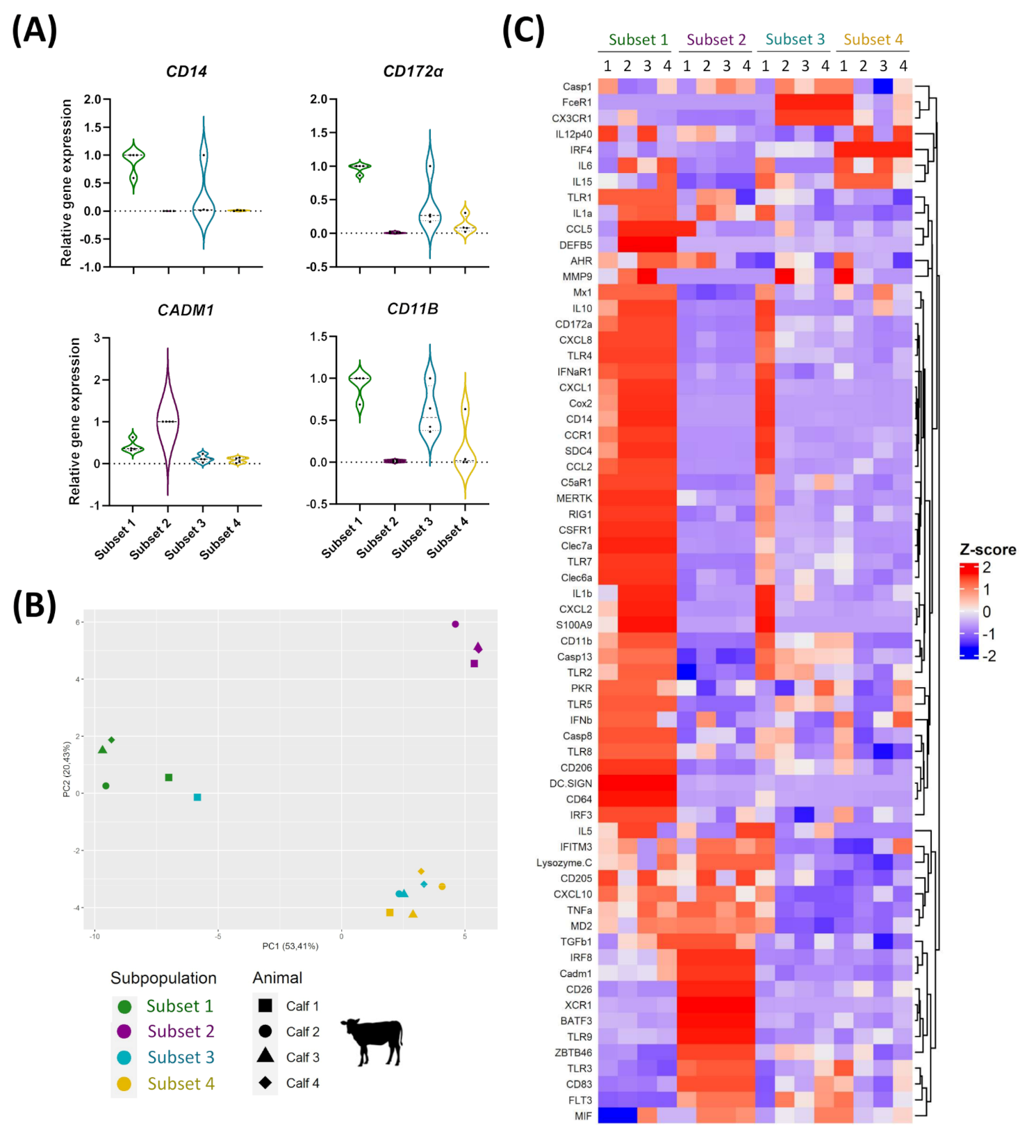
**

**Supplementary File 6. Transcriptomic analyses of mononuclear phagocyte subsets in the ileal Peyer’s patch of calf.** Quantitative RT-PCR with the FLUIDIGM® method were performed on mRNA from the four sorted ileal mononuclear phagocyte (MP) subsets of 10-day-old calves (n = 4) to assess gene expression. Complete list of the genes analyzed and corresponding primers is provided as Supplementary Table 1. Gene expression was defined by relative gene expression levels normalized to maximal expression across cell subsets, following normalization with three housekeeping genes (*HPRT*, *GAPDH*, *ACTB*) and 2e^-ΔCt^ value calculation. (**A**) Expression of the genes coding for the proteins used for cell sorting (*CD14*, *CD172α*, *CADM1*, *CD11B*) in each cell subset, represented by violin plots (median and quartiles). Each point corresponds to one animal. **(B)** Principal component analysis (PCA) represented by its first two dimensions with corresponding variances in percentage and performed on relative gene expression values. The 66 genes displayed in **(C)** were included in the PCA. Each point corresponds to data of one animal, with one point form per individual animal. **(C)** MP subset-specific gene transcription represented by heatmaps of the Z-score normalized relative gene expression values for each gene analyzed in the four MP subsets, with hierarchical clustering of genes.


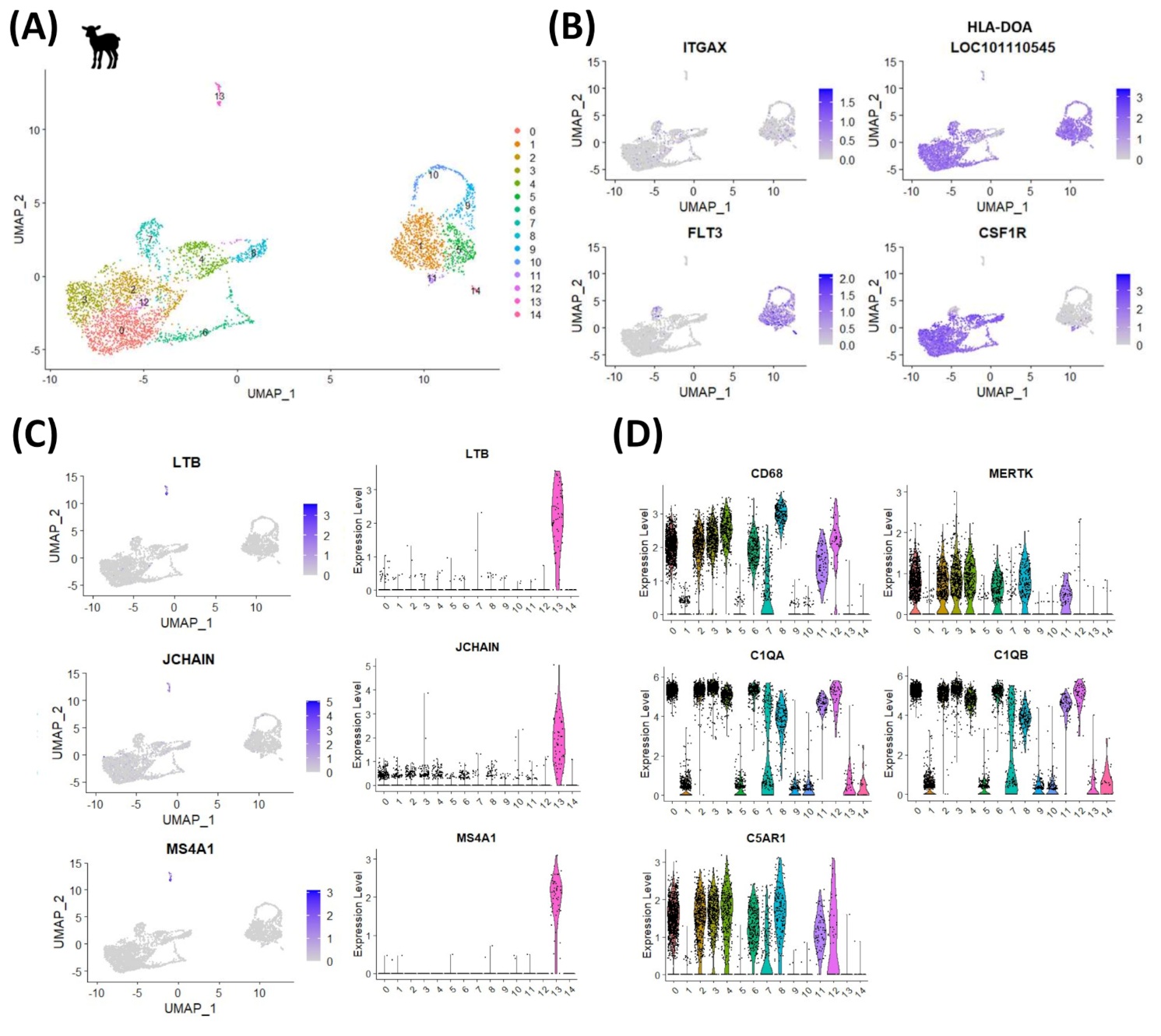


**Supplementary File 7. Initial characterization of intestinal mononuclear phagocytes in lamb by scRNA-sequencing.** Mononuclear phagocytes (MP) from the ileal Peyer’s patch of a 10-day-old lamb, identified as CD11c^+^MHCII^+^ cells, were sorted by flow cytometry and subjected to 10X Genomics scRNA-seq. **(A)** Data from 10 000 MP cells were analyzed and clustering was performed with a resolution of 0.5, resulting in 15 distinct clusters visualized by UMAP plot. **(B)** UMAP plots showing the expression of signature genes for MP (*ITGAX* (*CD11C*) and *OVA-DOA* (*MHCII*)), dendritic (*FLT3)* and monocytic cells (*CSF1R)*. **(C)** UMAP and Violin plots showing the expression of signature genes for B lymphocytes (*LTB, JCHAIN and MS4A1)* across all clusters. Expression levels are visualized from low expression (grey) to high expression (blue). **(D)** Violin plots showing the expression of supplementary genes characterizing monocytic cells (*CD68*, *MERTK*, *C1QA*, *C1QB*, *C5AR1*) across all clusters. Complete lists of the top 100 differentially expressed genes in the 15 clusters are given in **Supplementary Table 2**.


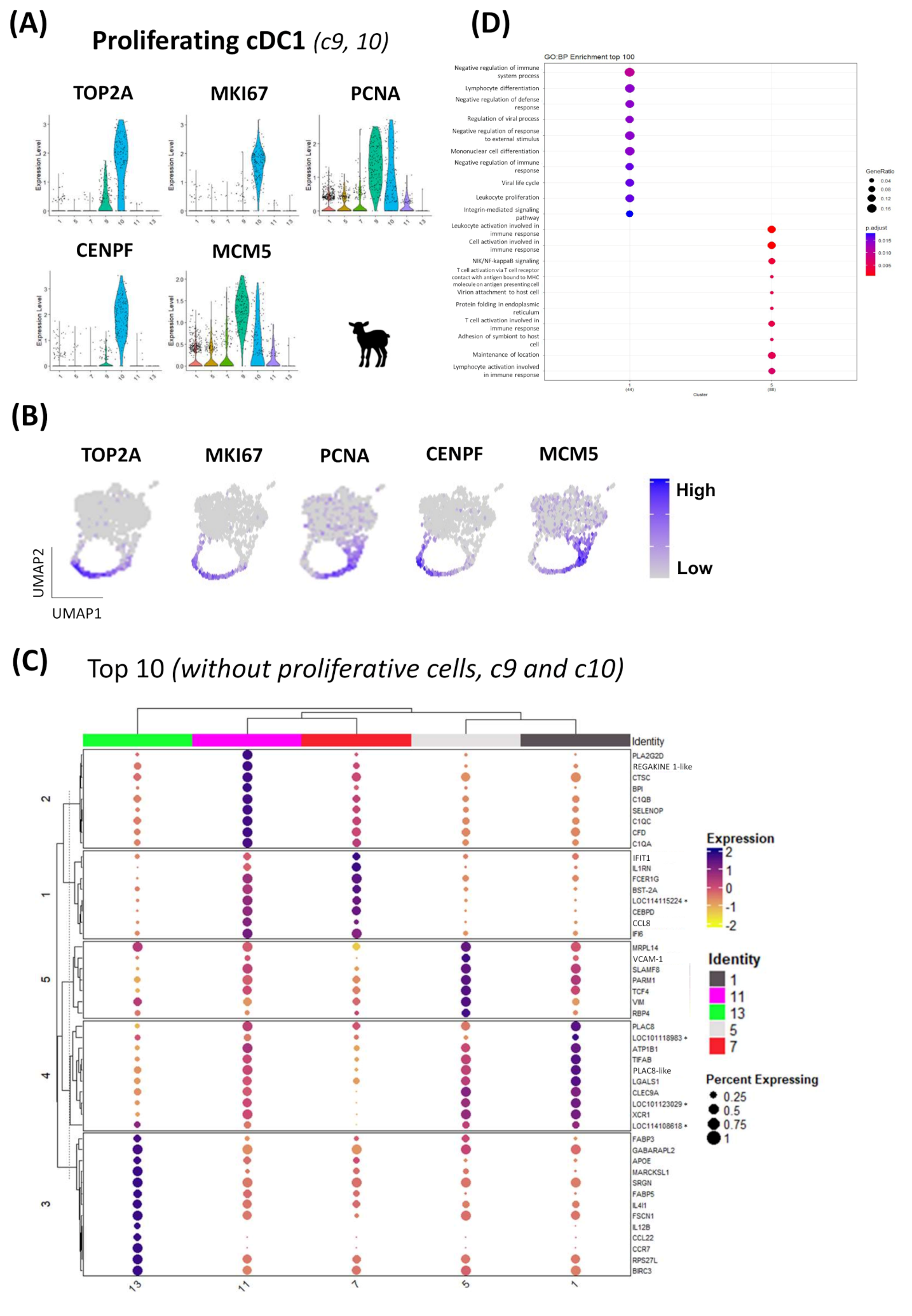


**Supplementary File 8. Comparison and characterization of intestinal dendritic cells in lamb by scRNA-sequencing.** Violin **(A)** and UMAP plots **(B)** showing the expression of signature genes for proliferating cells (*TOP2A*, *MKI67*, *CENPF*, *MCM5*, *PCNA;* c9 and 10) for each dendritic cell (DC) cluster. Expression levels are visualized from low expression (grey) to high expression (blue). **(C)** Dot plot showing the expression of the top 10 differentially expressed genes (lowest p_val_adj, highest avg_log2FC) for non-proliferative DC clusters (c1, 5, 7, 11 and 13), as determined by Seurat’s FindAllMarkers function. Complete gene lists are given in **Supplementary Table 5**. **(D)** Dot plot resulting from the gene ontology (GO) enrichment analysis of the top 100 differentially expressed genes between c1 and c5 of cDC1 (biological process) and showing the top 10 GO-terms significantly (p<0.05) enriched in c1 and c5, with their respective number of genes enriched in indicated below. Gene list is given in **Supplementary Table 6**. * Unknown annotations.


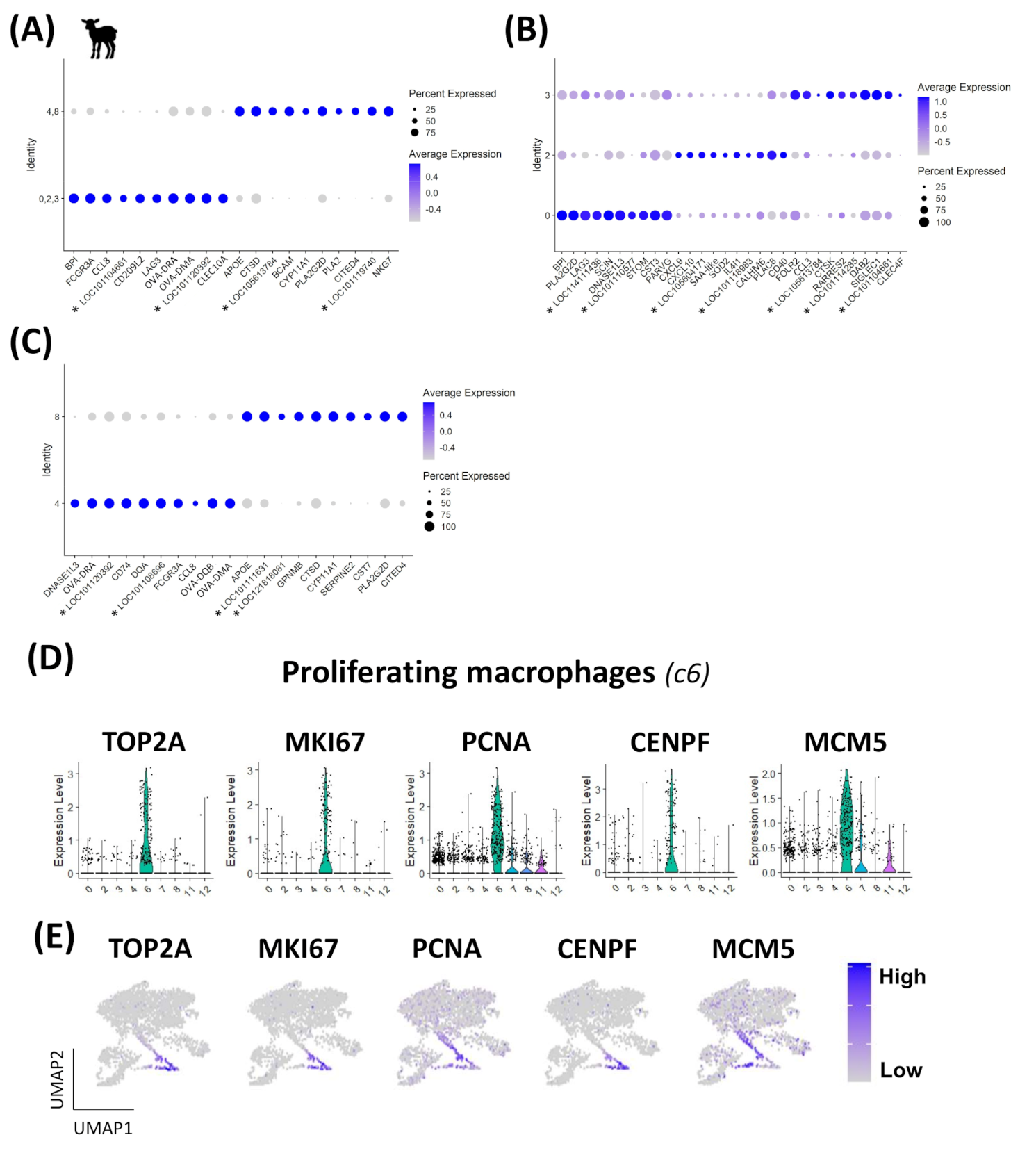


**Supplementary File 9. Comparison and characterization of intestinal monocytic cells in lamb by scRNA-sequencing.** **(A-C)** Top 10 differentially expressed genes (lowest p_val_adj, highest avg_log2FC) between mature MAC-like clusters (c0&2&3) and monocyte/early-MAC-like clusters (c4&8) **(A)**, between mature MAC-like clusters (c0, 2 and 3) **(B)** and between monocyte-like (c8) and early-MAC-like clusters (c4) **(C)**, as determined by Seurat’s FindAllMarkers function. Complete gene lists are given in **Supplementary Tables 8, 9 and 10**. Violin **(D)** UMAP plots **(E)** showing the expression of signature genes for proliferating cells (*TOP2A*, *MKI67*, *CENPF*, *MCM5*, *PCNA;* c6) for each monocytic cell cluster. Expression levels are visualized from low expression (grey) to high expression (blue). * genes of unknown function


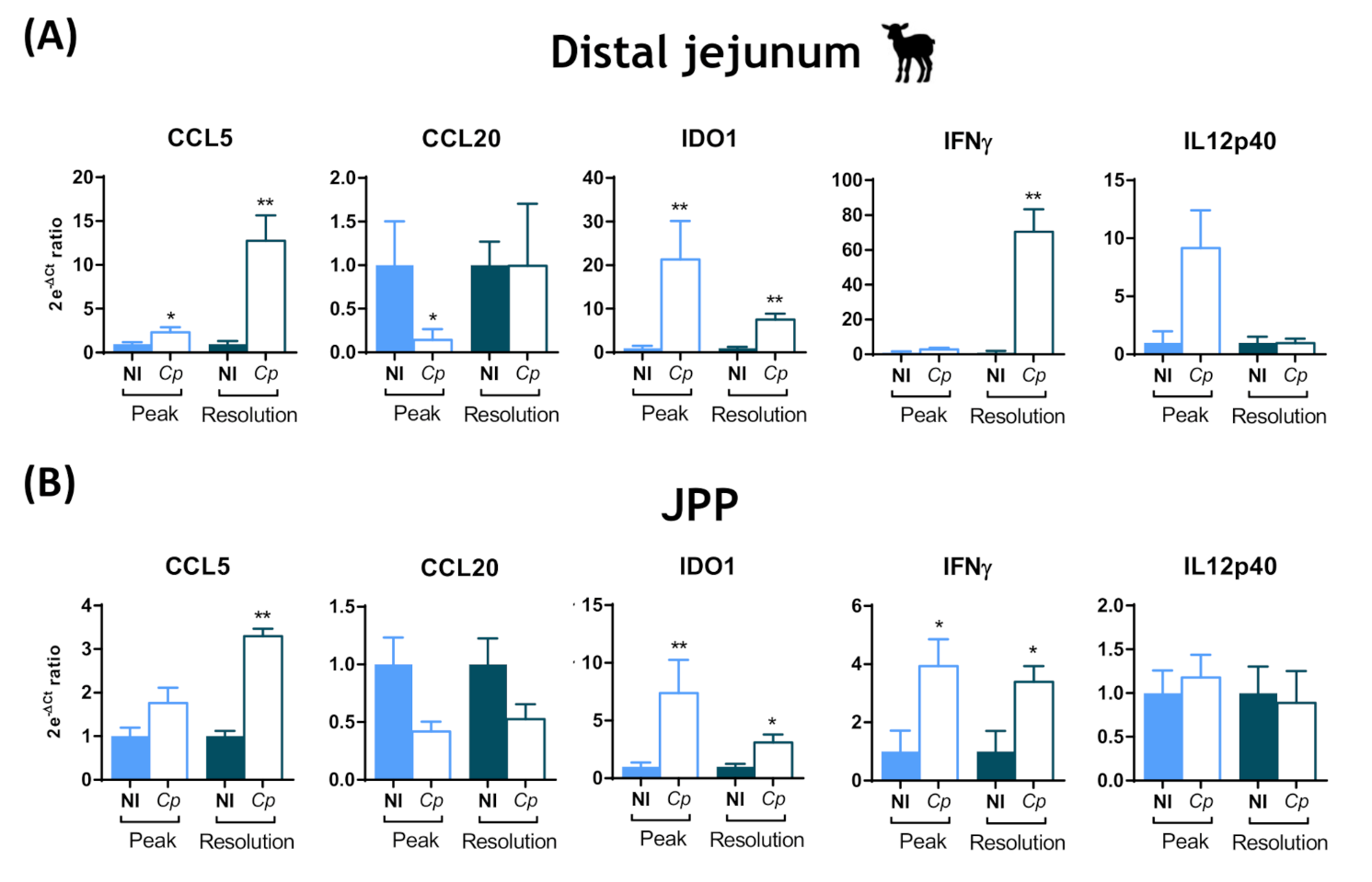


**Supplementary File 10.** **Immune responses in the distal jejunum and jejunal Peyer’s patches of lambs during *Cryptosporidium parvum* infection.** Three-day-old lambs were infected or not (NI) with 2.10^6^ oocysts of *Cryptosporidium parvum* (*Cp*) by oral route within two independent experimentations (n = 6-7/group/experimentation) and their distal jejunum and jejunal Peyer’s patches (JPP) were sampled at 6 days post infection (dpi) (peak of infection) or 11-12 dpi (resolution of infection). Intestinal tissues were processed for total RNA extraction and mRNA expression of *CCL5*, *CCL20*, *IFNγ*, *IL12p40* and *IDO1* genes were analyzed by RT-qPCR with the FLUIDIGM® method in the distal jejunum **(A)** and JPP **(B)**. Data are expressed as the ratio between the 2e^-ΔCt^ values of individuals and the mean of 2e^-ΔCt^ values of non-infected lambs (mean), following normalization with three reference genes (*hprt*, *gapdh*, *actb*), with each dot corresponding to one animal. « NI » for « Non infected ». Statistical analyses were performed out by Mann-Whitney non-parametric test to compare the medians of gene expression between infected and non-infected animals; statistical significance was determined by a P-value<0.05 (*P<0.05, **P<0.01).


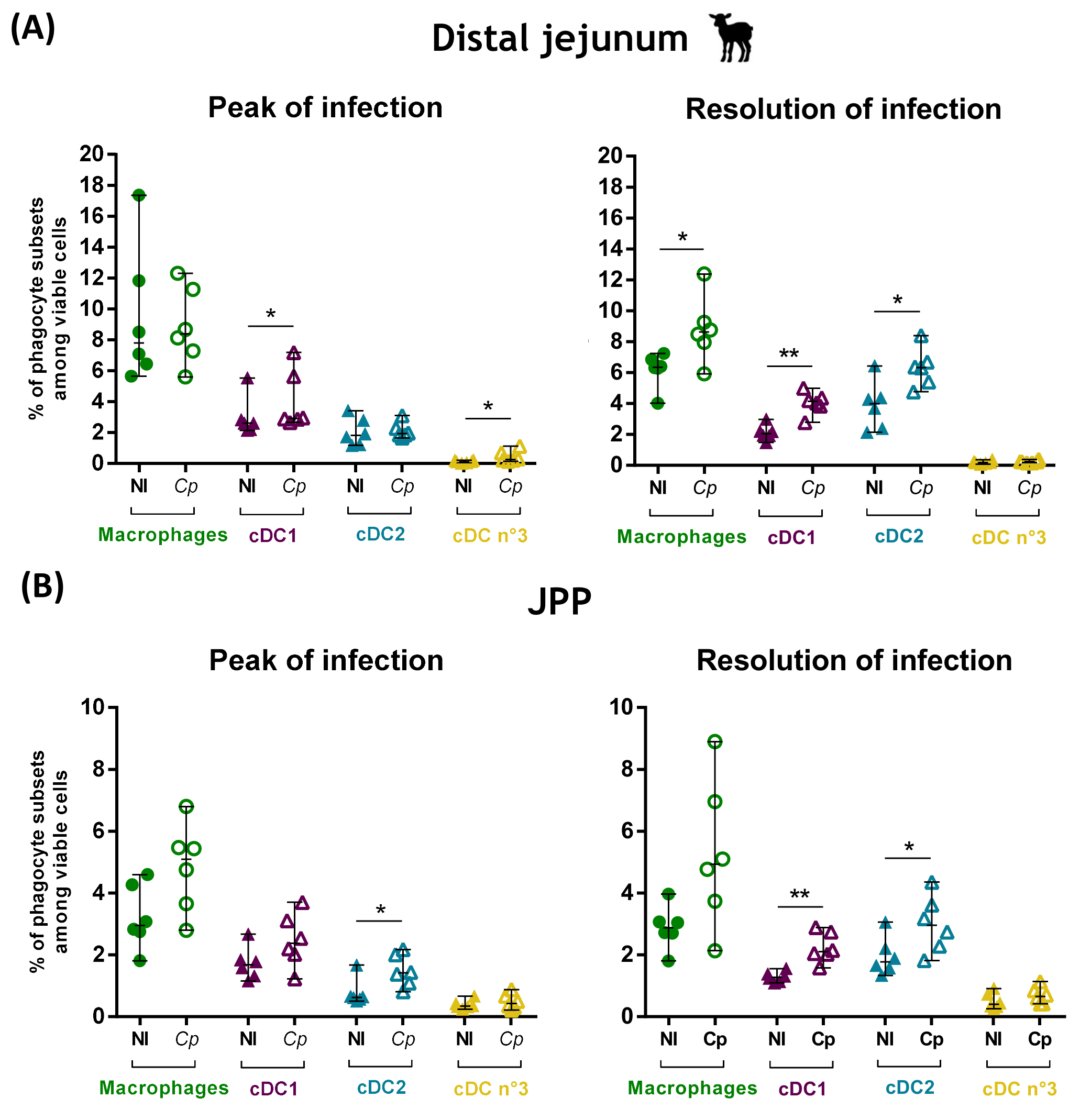


**Supplementary File 11. Mononuclear phagocyte recruitment/proportion in the distal jejunum and jejunal Peyer’s patches of lambs during *Cryptosporidium parvum* infection.** Three-day-old lambs were infected or not (NI) with 2.10^6^ oocysts of *Cryptosporidium parvum* (*Cp*) by oral route within two independent experimentations (n = 6-7/group/experimentation) and their distal jejunum and jejunal Peyer’s patches (JPP) were sampled at 6 days post infection (dpi) (peak of infection) or 11-12 dpi (resolution of infection). Proportions of macrophages (green), cDC1 (purple), cDC2 (blue) and cDC n°3 (yellow) in the distal jejunum **(A)** and JPP **(B)** were analyzed by flow cytometry following mechanical and enzymatic dissociations of intestinal tissues, isolation of total isolated intestinal cells and staining. Data are expressed in percentage among viable cells (median ± range) with each dot corresponding to one animal. « NI » for « Non infected ». Statistical analyses were performed out by Mann-Whitney non-parametric test to compare the medians of proportions of mononuclear phagocyte subsets between infected and non-infected animals; statistical significance was determined by a P-value<0.05 (*P<0.05, **P<0.01).


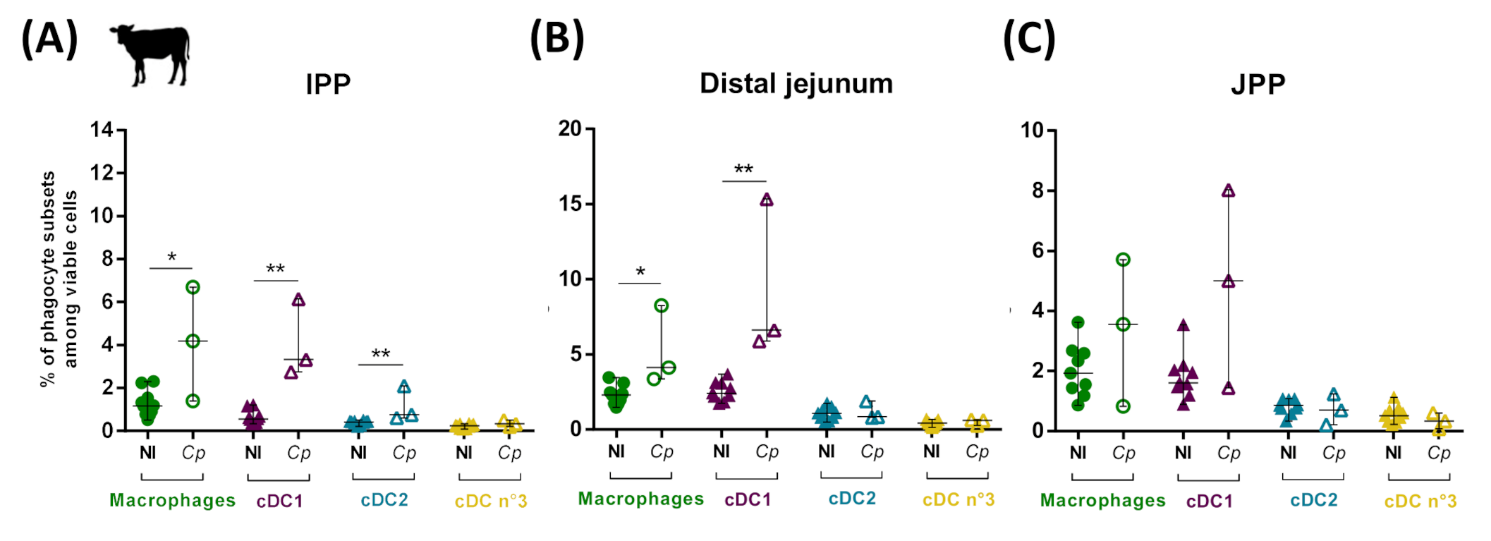


**Supplementary File 12. Mononuclear phagocyte recruitment/proportion in the intestine of calves during *Cryptosporidium parvum* infection.** Day-old calves were infected or not (NI) with 8.10^6^ oocysts of *Cryptosporidium parvum* (*Cp*) by oral route (n = 9-10 for the non-infected group and n = 3 for the infected one) and their ileal Peyer’s patches (IPP), distal jejunum and jejunal Peyer’s patches (JPP) were sampled at 11-12 days post infection (dpi) (resolution of infection). Proportions of macrophages (green), cDC1 (purple), cDC2 (blue) and cDC n°3 (yellow) in the IPP **(A)**, distal jejunum **(B)** and JPP **(C)** were analyzed by flow cytometry following mechanical and enzymatic dissociations of intestinal tissues, isolation of total isolated intestinal cells and staining. Data are expressed in percentage among viable cells (median ± range) with each dot corresponding to one animal. « NI » for « Non infected ». Statistical analyses were performed out by Mann-Whitney non-parametric test to compare the medians of proportions of mononuclear phagocyte subsets between infected and non-infected animals; statistical significance was determined by a P-value<0.05 (*P<0.05, **P<0.01).
